# Nonlinear Electrical Conduction in Polymer Composites for Field Grading in High-Voltage Applications: A Review

**DOI:** 10.3390/polym13091370

**Published:** 2021-04-22

**Authors:** Alejandro Can-Ortiz, Lionel Laudebat, Zarel Valdez-Nava, Sombel Diaham

**Affiliations:** 1 LAPLACE, Université de Toulouse, CNRS, INPT, UPS, 31062 Toulouse, France; can@laplace.univ-tlse.Fr (A.C.-O.); laudebat@laplace.univ-tlse.fr (L.L.); diaham@laplace.univ-tlse.Fr (S.D.); 2 Institut National Universitaire Champollion, Université de Toulouse, Place de Verdun, 81012 Albi, France

**Keywords:** nonlinear polymer composites, high voltage applications, field grading material, nonlinear conductivity, non homogenous polymer composites

## Abstract

Applications of polymeric materials in electrical engineering increasingly require improvements in operating voltages, performance, reliability, and size reduction. However, the resulting increase on the electric field in electrical systems can prevent achieving these goals. Polymer composites, functionalized with conductive or semiconductive particles, can allow us to reduce the electric field, thus grading the field within the system. In this paper, a comprehensive review of field-grading materials, their properties, and recent developments and applications is provided to realize high-performance high-voltage engineering applications.

## 1. Introduction

The total electrical power consumption of the world increases with the number of electrical systems. Thus, the efficacy of electrical systems must be increased by improving their design, performance, and reliability. Size reduction impacts their weight–to–power relationship and in turn, their energy density. On highly constrained systems, size reduction affects the applied electric field as well as the operating temperature. To address the limitations of currently available materials, developing new materials that can intrinsically withstand or reduce constraints or modifying their configurations or properties is necessary.

In most electrical systems, the electric field has a nonhomogeneous distribution, which can involve electric field concentrations that reduce the lifetime of the material [1,2,3]. In the following sections, electrical stress concentration issues and currently available solutions are presented. Among them, polymer composites that are used as electric field-grading materials provide electric field concentration reduction, improving the lifetime of the electrical system. Thus, we describe factors that influence the electrical properties of isotropic composites and anisotropic materials with graded electrical conductivities. Finally, the advantages of using polymer composites with graded electrical conductivities in high-voltage (HV) engineering applications are presented.

## 2. Electric-Field Grading in HV Applications

An electrical system is usually made up of multiple elements or devices connected in a serial arrangement. A failure in any of them may cause the failure of the whole system [4]. In HV applications such as rotating machines, the insulating material is one of the weakest elements of the electrical system, leading to reliability loss [5]. In electrical machines such as hydrogenerators, failure due to insulation damage is higher than 50% [6,7].

It is known that the rate of aging, the rate at which the essential properties of insulation reduces, is not only a function of the magnitude, but it also depends on the nature of the applied stress [8,9,10]. The most important stress types to which insulation is subjected are electrical, thermal, and mechanical stresses [8,10,11]. When high electric stress is applied to most polymers, charges are injected and accumulated within them [12], distorting the internal electric field distribution. Consequently, insulation degrades because of space charge accumulation, partial discharge (PD), and electrical treeing across the whole material, which may lead to breakdown [13,14].

**Figure 1:**
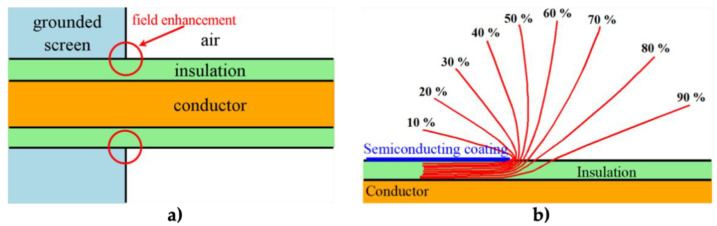
Insulated conductors passing through a grounded metallic screen at HV. (**a**) Typical configuration. © 2021 IEEE. Reprinted, with permission, from Ref. [20]. (**b**) Equipotential and stress distribution due to the termination of the semiconducting coating. © 2021 IEEE. Reprinted, with permission, from Ref. [21].

The main polymers used for insulation in HV applications include epoxy resin, ethylene propylene diene monomer (EPDM), ethylene propylene monomer, silicone rubber (SR), EPDM/silicone rubber alloy, and polyethylene (PE) [12,15]. Low-density PE (LDPE) and cross-linked PE are mainly used in power cables for HV direct current (HVDC) applications. However, the development of a nonhomogeneous distribution of space charge or polarization under a high DC voltage can dramatically increase the internal electric field, depending on the material structure, additives, impurities, applied field, and temperature [16,17]. Another problem with cables in HV applications is the failure of connectors and cable termination. Cable joints and terminations constitute the main points of weaknesses of power systems (more than 95%) [18,19,20]. The insulated electrical conductor is placed in a grounded screen, and near the screen, field enhancement occurs at the sharp edge, where equipotential lines diverge (Figure 1) [20,21].

Figure 1 shows the presence of air inducing a triple point at the metal/dielectric/air intersection [20]. A dramatic electric field enhancement causes PD, flashover, and breakdown [16,22,23].

Besides, electrical insulation of HV rotating machines is particularly important in generators and motors to reduce manufacturing and maintenance costs [9]. One of the main polymers used in these applications is epoxy resin (EP) [24]. The stator insulating system may involve defects created by combined thermal, mechanical, electrical, and environmental stresses, resulting in gradual deterioration [13,25,26,27]. In particular, electrical stress can cause PDs in voids and cavities, thereby eroding insulating materials and leading to electrical treeing [13,25]. At the end of the laminated core, the electrical insulation of the stator end windings is stressed tangentially at the boundary surfaces due to the concentration of the electric field. The air near the end-turn insulation breaks down, leading to surface corona discharges. Not only corona discharges but also internal discharges can directly accelerate the deterioration of polymers due to highly localized electrical stress concentrations and ultimately, lead to the failure of the insulation material [28,29].

**Figure 2:**
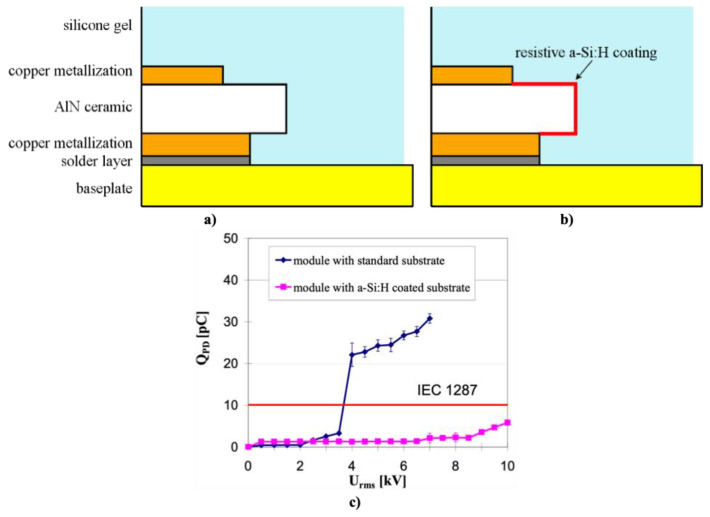
IGBT modules (**a**) without (**b**) with resistive a-Si:H coating. (**c**) Comparison of the PD of both modules. © 2021 IEEE. Reprinted, with permission, from Ref. [3].

High-power insulated-gate bipolar transistor (IGBT) modules are key components in industrial, traction, and HVDC transmission applications [30]. Although the failure rate of power modules has been drastically reduced, IGBT modules are still one of the most fragile components regarding system reliability [30]. The increasing demand for larger power densities requires increasing the voltage, temperature, and frequency [31]. Due to the high voltage, modules have a strong electric field reinforcement in the insulation media that causes dielectric breakdown [32]. The main component affected is the metalized aluminum nitride ceramic substrate embedded in a silicone gel (Figure 2). PDs or even breakdown of the insulation can occur due to the electric field concentration at the edges of the metalized substrate that can exceed the dielectric strength of the silicone gel [3,33,34].

Thus, one of the key subjects in HV engineering is the control of the electric field strength. The aim is to achieve the lowest possible electrical field strength at a fixed system voltage [22]. Stress control methods fall into two main approaches: (i) *capacitive stress control*, for example, geometrical electrode grading by controlling the shape of conducting parts, refractive grading by high-permittivity materials, and impedance stress control by applying layers with specific resistivity to the screen cut and cable insulation; (ii) *resistive stress control* using materials with specific current–field characteristics [22,35,36]. Eigner and Semino [22] reported various technologies used for stress control, and their results are presented hereafter.

**Figure 3:**
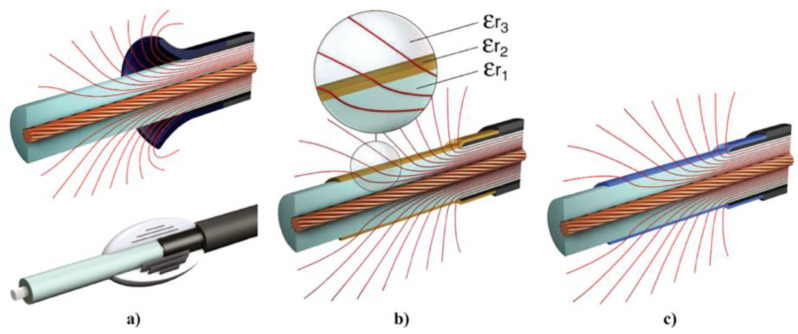
Various technologies used for stress control in cable termination: (**a**) Geometric stress control (up) with conductive layers (down), (**b**) refractive stress control (insulation ($\varepsilon_{r_{1}}$), control ($\varepsilon_{r_{2}}$ ), and surrounding ($\varepsilon_{r_{3}}$ ) materials), and (**c**) impedance stress control. © 2021 IEEE. Reprinted, with permission from [22].

Geometric stress control has two types (Figure 3a): The first type involves reducing the electric field by optimizing the geometry of the electrodes since the equipotential lines follow the electrode shape. Two main shapes can be used, namely, Borda and Rogowski profiles, where both types simultaneously allow compact design and optimized stress control [22]. The second type consists of technologies based on conductive layers that are applied in the area with the highest electrical stress, and stress control is based on the positions and dimensions of the insulating and conductive layers. Typical conductive materials include graphite, conductive polymeric and carbon black tapes, and metallic foils [22].

In refractive stress control (Figure 3b), nonconducting materials with high permittivity are used. It can be applied by wrapping tapes around areas with high electrical stress or incorporating refractive materials in base materials, such as PE, silicone, or EPDM. This method is based on the fact that the permittivity of the refractive material ($\varepsilon_{r_{2}}$) is always much higher than those of the insulator ($\varepsilon_{r_{1}}$) and environment ($\varepsilon_{r_{3}}$). The ratio of electrical stresses in two different materials ($\varepsilon_{r_{1}}$ and $\varepsilon_{r_{3}}$) can be adjusted by inserting a material with high permittivity between them ($\varepsilon_{r_{2}}$), and high electrical stresses can be reduced to noncritical values. The major limitation of this technique is the dielectric loss in the refractive material. This loss increases with the volume and permittivity of the refracting material. It is also necessary to properly design the insulation system for adequately transfer heat to avoid local overheating, especially at higher frequencies [22].

**Figure 4:**
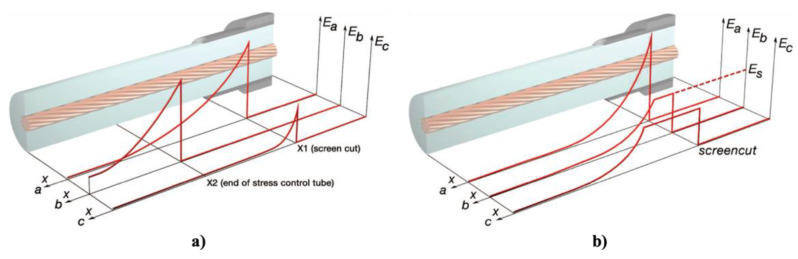
Electric stress distribution as a function of the position between the ground and HV electrodes (**a**) with impedance stress control and (**b**) nonlinear stress control. © 2021 IEEE. Reprinted, with permission, from [22].

Impedance stress control (Figure 3c) consists of applying layers with specific electrical resistivities to the screen cut and cable insulation. These layers are commonly deposited using shrinkable tubes or patches. For this method, when the impedance is chosen, there are three options. The first one is to use an insulating material (Z = ∞). As a consequence, the material is at the full potential leading to a steep slope of voltage at the screen cut of the power cable. The electrical field strength is thus very high at this location (Figure 4, curve a). A highly insulating material is undesired because the electrical stress remains at the surface. The second option is to apply a material with high conductivity (Z = 0). However, it is also undesired because it does not reduce the electrical field strength at the screen cut (X1) but moves the maximum to the end of the stress control material (X2) (Figure 4a, curve b).

The last option is to apply a material with intermediate conductivity by placing this stress control material over the screen cut. Thus, the area with the highest field strength is covered and controlled. The electric field at the screen cut is reduced to an acceptable working value (Figure 4a, curve c). This option prevents the accelerated aging of cable insulation and the accessory itself. The main advantage of this stress control system is its slim and compact design, which is independent of the size of the power cable [22].

Finally, nonlinear stress control (Figure 4b) uses materials whose resistivity varies with the applied voltage [37]. These materials can be developed using a base material, which is usually an insulating polymer with an additive that provides nonlinear functionalities. Common additives used for corona protection for motor stator bars and cables are SiC, ZnO, carbon black, or blends of different oxides such as BaTiO_3_, TiO_2_, SiO_2_, Fe_3_O_4_, and mica [18,22,35]. One important advantage of using nonlinear field-grading materials is the compact design of cable accessories, which makes them easy to install and thus reduces costs [38]. As can be observed in Figure 4b, without any stress control, the highest electric field appears at the screen cut, increasing the probability of triggering PD activitiy in this region. By placing a nonlinear stress control tube over the screen cut, the electrical stress can be limited to a certain level ($E_{S}$) (Figure 4b, curve b). This level does not change, even at higher voltages (Figure 4b, curve c) [22].

One example for impedance stress control was reported for IGBT modules. The edge of the ceramic was coated with a high-impedance layer, which electrically connects the top copper metallization with the bottom one, thus leading to a very low electric current between the collector and ground [3,33]. It is reported that the electric current homogenizes the electric field strength at the edge of AlN coated with high-impedance doped amorphous silicon (-Si:H) (Figure 2) [3]. As shown in Figure 2c, coated AlN substrates substantially increase the PD resistance of IGBT modules [3]. Finite element method (FEM) simulations showed that the application of a nonlinear resistive layer at the metallization edge of power electronic substrates significantly reduces the electric field at the critical triple point between the copper metallization, ceramic substrate, and silicone gel or polyimide encapsulation [33,39].

Some examples of nonlinear stress control have been reported for rotating machines. Nonlinear resistance tapes are the universal choice for stress-grading motor and generator coils [21]. Evaluating system performance by simulating the electric field and potential distributions in the overhang region showed that a highly conductive stress-grading tape reduces the maximum electric field at this region [40]. Usually, the main insulation is provided with outer corona protection applied by multilayer semiconducting paints or wrapping tapes [28]. Carbon-based semiconductive tapes and SiC-based nonlinear grading tapes have been widely used to control the field where the stator bar exits from the slot in form-wound large rotating machines [41]. The conductivity of the stress-grading tape depends on the electric field and is high at the end of the semiconductive armor tape, where the electric field is the highest and gradually decreases along the stress-grading tape. Consequently, it makes the electric field at the end of the armor tape more uniform.

In general, nonlinear resistive field-grading materials have a field-dependent conductivity, σ(E), which increases strongly from a low conductivity value, σ(0), to a high value in a narrow field region ΔE near $E_{b}$ [20,23,35]. The value of the switching field ($E_{b}$) indicates where field grading becomes active [35]. Efficient nonlinear resistive field grading needs a sufficiently large nonlinearity coefficient, α [35]:

(1) $$\alpha =\frac{d\ln\left(j\right)}{d\ln\left(E\right)}=1+\frac{d\ln\left(\sigma \right)}{d\ln\left(E\right)}$$

Nonlinear field grading implies that a conduction current flows in the field-grading material, leading to Joule heat production [35]. Thus, a reasonably designed nonlinear material should be active only in a small region or during short time intervals (impulses, surges) so that heating is noncritical [35].

**Figure 5:**
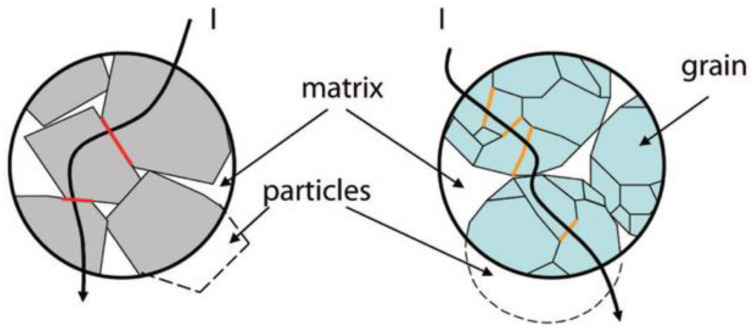
Schematic of the microstructures of field-grading materials based on SiC (**left**) and ZnO microvaristors (**right**). Arrow: possible current path; red: particle–particle contact responsible for the nonlinearity in SiC-based materials; orange: grain boundary responsible for the nonlinearity in microvaristor-based materials. © 2021 IEEE. Reprinted, with permission, from Ref. [42].

Donzel et al. proposed a model to describe the origin of nonlinearity in composites, where the filler content must be greater than the percolation threshold so that particles form a continuous, percolating network to ensure conductivity [42]. Nonlinearity in composites originates from two sources: particle–particle contacts and intrinsic properties of filler particles (Figure 5). In the first category, the surfaces of the conducting or semiconducting filler particles are either surrounded by a thin layer of oxide or another material, or they contain surface (interface) states leading to surface charging and band bending, similar to Schottky barriers in conventional semiconductors [42]. A combination of the two effects can also exist. Changes in the band diagram at the particle–particle contacts lead to a nonlinear transport for electrons or holes passing from one particle to the next. Transport occurs either by hopping, tunneling, thermal activation over potential barriers, or combined transport mechanisms. For non-oxide filler particles such as SiC, C, and Al, nonlinearity occurs due to the spontaneous formation of thin interfacial oxide layers in polymer composites. For particles of semiconducting oxides, surfaces are chemically more inert. Nonlinear resistive transport can be due to surface band bending caused by surface charging that forms back-to-back Schottky barriers at particle contacts. In more complex composite mixtures containing SiC and carbon black fillers, hetero-contacts such as C/SiC may also form and can reduce the percolation threshold for current transport [42]. For materials where nonlinearity stems from particle–particle contacts, nonlinearities are usually moderate with nonlinearity coefficients (α) in the range 3–8 [42]. In the second category, a fundamentally different approach is taken. The nonlinearity is an intrinsic property of the particle alone, and the particle–particle contact resistance is low. Microvaristors [42] and microvaristor-based composites have much higher nonlinearity coefficients, typically between 20 and 30 [42].

## 3. State-of-the-Art of Particle Properties

To obtain nonlinear properties required in various HV applications presented above, various fillers have been used to modify the electrical properties of composites. The final composite conductivity depends on the filler type, their physicochemical characteristics, size and shape, and interaction with the matrix. Particles used in nonlinear composites and their characteristics are presented in Table 1, Table 2 and Table 3 for ZnO, SiC, and carbon allotropes, respectively.

As can be seen from Table 1, Table 2 and Table 3, fillers possess various characteristics, and each parameter has a different influence on the final properties of composites. Their impacts are discussed in the next section.

## 4. Polymer Composites with a Single Particle Type

### 4.1. ZnO-Based Composites

ZnO is one of the most used filler materials for nonlinear conduction applications. At low electric fields, it exhibits a linear current–voltage relationship. Above a certain electric field, its conductivity starts to increase, characterized by a dramatic nonlinear increase in the current around the points with high voltage [57]. The final properties of the composite (switching field and nonlinearity coefficient) depend on the characteristics of the filler, such as the filler concentration, size, morphology, and composition. In the next sections, the influences of these factors are reviewed.

#### 4.1.1. Effect of Filler Concentration

The filler concentration has an important influence on the final properties of composites. For example, Auckland et al. [77,78] fabricated composites of polyester with 8, 14, and 30 vol.% of ZnO varistors. Composites with 8 vol.% ZnO produced small currents comparable with those of the unfilled polyester. Composites with 14 vol.% ZnO showed nonlinear conduction, where the electrical conduction rapidly increased above 0.1 MV/cm [78]. Finally, with 30 vol.% ZnO, the concentration was so high that the conduction changed significantly, even at low fields [77]. Tavernier et al. [43,44] studied composites of polyester and 10, 20, 30, 40, and 50 wt.% ZnO fillers (~6 µm granule size). When the filler concentration was 30 wt.%, the conductivity in the filled resin had a nonlinear relationship with the electric field. The conductivity increased with the electric field, and the dependence was higher when the filler concentration increased [43,44].

**Figure 6:**
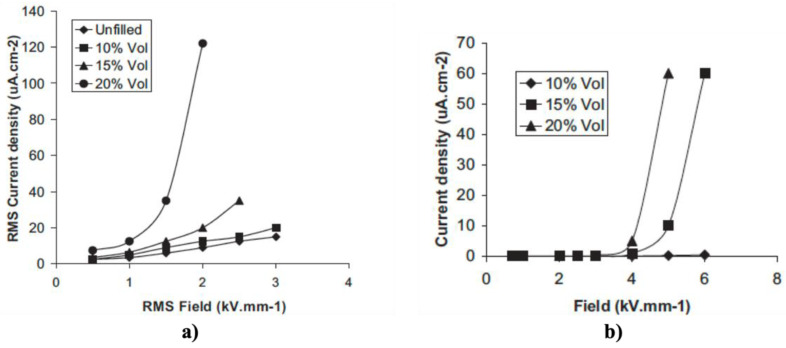
Current density versus electrical field strength for ZnO-filled epoxy resin. (**a**) AC and (**b**) DC. Republished with permission of Institution of Engineering and Technology (IET), from [45], © 2021; permission conveyed through Copyright Clearance Center, Inc.

Varlow et al. [45] reported composites of epoxy resin (MY750 resin, HY917 hardener, and DY073 accelerator) and 10, 15, and 20 vol.% ZnO (undoped, laboratory-grade, 99.9% purity, 1 μm). Their results are presented in Figure 6.

The AC conductivity in composites with 10 vol.% ZnO remained linear and only marginally higher than that of the unfilled resin [45]. For 15 vol.% ZnO, the nonlinear behavior was observed above a threshold field (switching field) of about 2 kV/mm [45]. Composites with 20 vol.% ZnO showed a much larger nonlinear conduction behavior with a lower threshold field (around 1 kV/mm) than that with 15 vol.% ZnO [45]. The same behavior was observed in DC characterization. Composites with 10 vol.% ZnO did not present a nonlinear behavior. As the ZnO concentration increased to 15 vol.%, the behavior of the samples became nonlinear, and the nonlinearity became even more pronounced as the volume fraction of ZnO increased to 20% [45].

**Figure 7:**
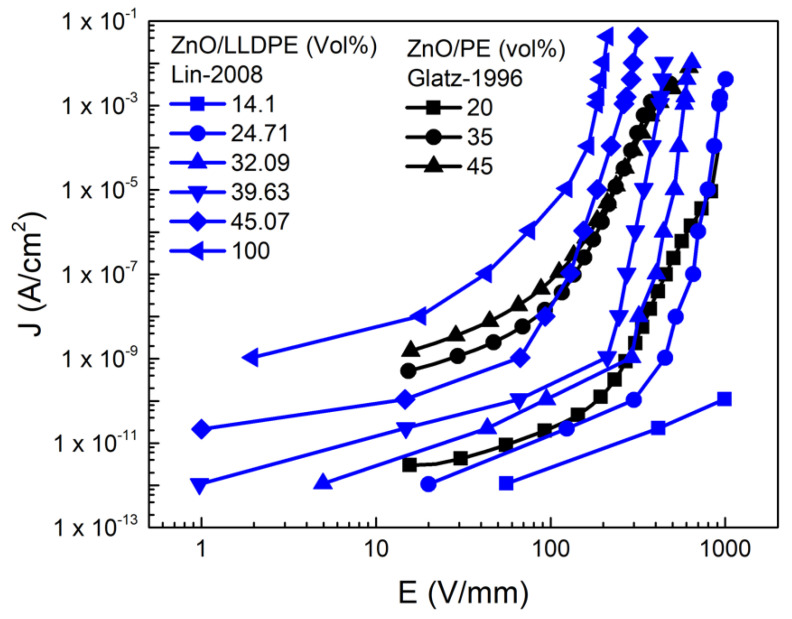
*J–E* characterization of LLDPE and PE composites with varying ZnO filler concentrations. Data extracted from Refs. [50,51].

Auckland et al. [48] studied composites of LDPE and 10, 15, 20, 25, and 30 wt.% ZnO (diameter of 1 μm). They reported a linear relationship between the current density (*J*) and electric field (*E*) for LDPE. A nonlinear *J*–*E* relationship was observed for composites with above 10 wt.% ZnO due to the abrupt increase in *J* beyond a critical threshold field, and the switching field decreased as the ZnO concentration increased [48]. Glatz-Reichenbach et al. [51] worked with PE and commercially available doped ZnO-varistor powder (spherical, 60–160 μm), ranging the filler concentration between 20 and 50 vol.%; Lin et al. [50] reported the results for linear low-density polyethylene (LLDPE) using ZnO with a diameter between 0.1 and 1 μm and concentrations varying from 14.1 to 100 vol.%. The fillers were obtained using Bi_2_O_3_ + Sb_2_O_3_ as boundary formers and CoO + MnO as conductivity enhancers [50]. The results of both papers are presented in Figure 7 and Table 4.

Figure 7 shows that the composites exhibited nonlinear conduction. The current density (*J*) increased when the electric field (*E*) exceeded *E_b_* for each composite. The nonlinear parameters (Table 4) depend on the filler concentration. *E_b_* decreased when the filler concentration increased. In the case of PE composites, α was independent of the filler concentration. For composites of LLDPE, α decreased when the filler concentration increased. The difference in the behavior of different polymers can be explained by differences in the contact resistivity of fillers [50].

**Figure 8:**
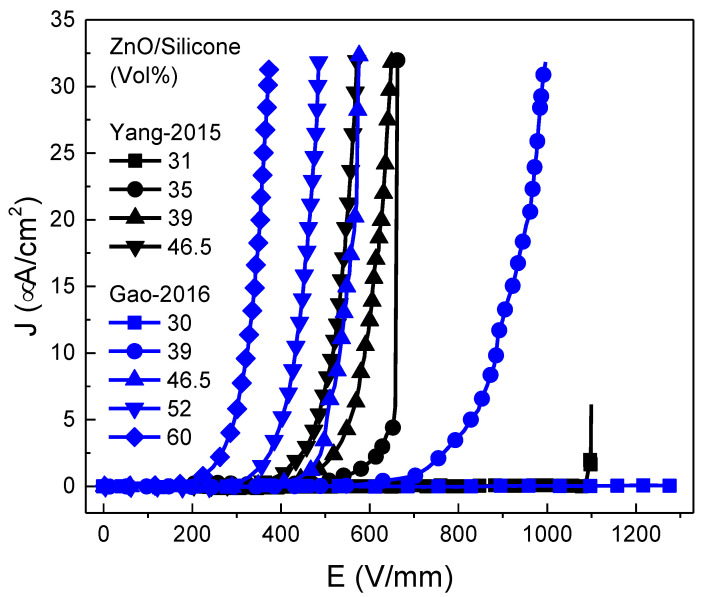
*J–E* characterization of silicone rubber composites with varying ZnO filler concentrations. Data extracted from [54,55].

Moreover, nonlinear conduction was reported for composites of ZnO/silicone rubber [54,55]. Microspherical ZnO varistors were used as fillers (95 mol % ZnO + 1.0 mol % Bi_2_O_3_ + 0.5 mol % MnO_2_ + 1.0 mol % Co_2_O_3_ + 0.4 mol % Cr_2_O_3_ + 1 mol % Sb_2_O_3_ + 1.0 mol % SiO_2_ + 0.1 mol % Al_2_O_3_) [54,55]. Yang et al. [54] reported results for fillers with a diameter between 100 and125 μm and filler concentrations of 31, 35, 39, and 46.5 vol.%. Gao et al. [55] reported results for fillers with average sizes of microspheres and grains of 120 and 9.5 μm, respectively, and filler concentrations between 30 and 60 vol.%. The results for both papers are presented in Figure 8.

Silicone composites filled with ZnO microvaristors possessed nonlinear conduction when the filler concentration was above the percolation threshold (39 vol.%), and the switching field was controlled by changing the filler concentration (Figure 8) [54,55]. As the filler concentration increased, the nonlinearity occurred earlier, indicating a lower switching field [54]. The nonlinear coefficients for these composites were 12.5, 15.8, 17.1, and 19.0 for 39, 46.5, 52, and 60 vol.% ZnO microvaristors, respectively [55].

**Figure 9:**
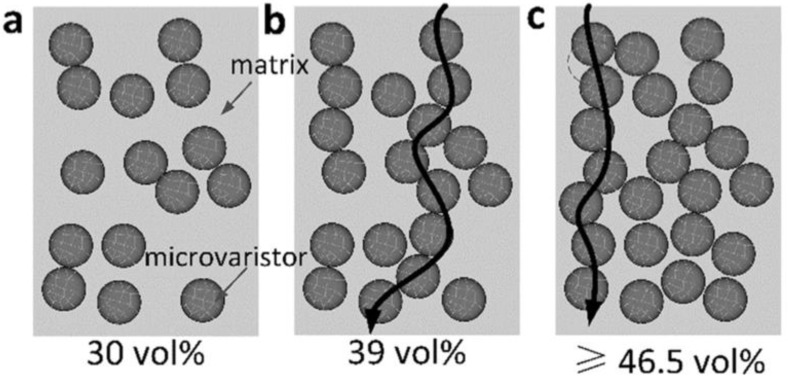
Schematic of filler distribution in ZnO/Silicone rubber composites with (**a**) 30 vol.%, (**b**) 39 vol.%, (**c**) ≥46.5 vol.% ZnO. Reprinted from Ref. [55], Copyright 2016, with permission from Elsevier.

These studies show that the nonlinear conductivity of composites strongly depends on the filler concentration. The switching field decreases when the filler concentration increases as the nonlinearity of composites depends on ZnO properties. The additives in ZnO create Schottky barriers at both sides of grain boundaries, and these barriers are responsible for the nonlinear characteristics of varistors [77,78]. Thus, it is possible to find nonlinear conductivity in the composites at sufficiently high filler concentrations [77,78]. Gao et al. [55] presented a schematic of filler distribution in ZnO microvaristor/rubber composites to explain the mechanism of nonlinear conductivity (Figure 9). It shows that no conduction paths were generated with low filler concentration (30 vol.%), which led to low conductivity (Figure 9a) [55]. However, when the filler concentration exceeded the percolation threshold, conduction paths leading to nonlinear conduction were formed (Figure 9b) [55]. In composites with relatively high filler concentration (Figure 9c), several conduction paths coexisted although the shortest path was used. Thus, the switching field dramatically decreased [55].

#### 4.1.2. Effect of Filler Shape and Size

**Figure 10:**
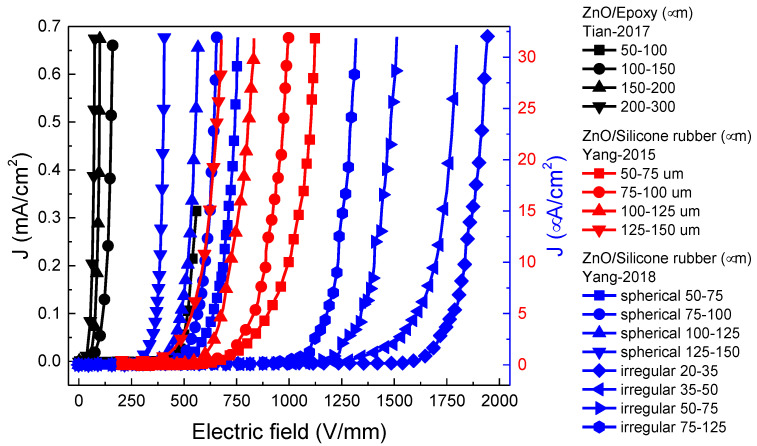
*J–E* characterization of composites with ZnO as a filler: effects of filler size and morphology. Data extracted from [46,52,54].

**Figure 11:**
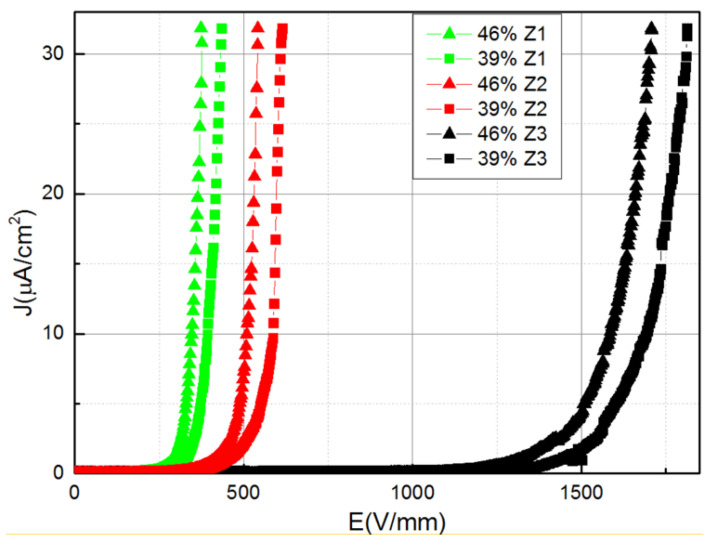
*J–E* characterization of Z1, Z2, and Z3 composites involving 46.5 vol.% and 39 vol.% filler concentration with filler diameters in the range 75–125 μm. © 2021 IEEE. Reprinted, with permission, from [56].

The filler size is another parameter that affects the properties of composites. The influence of the ZnO filler size on the nonlinear electrical properties of composites has been reported [46,52,54]. Tian et al. [46] studied this effect using epoxy and 20 vol.% of ZnO (99 mol % ZnO + 1 mol % K_2_CO_3_) with sizes ranging from 50 to 100, 100 to 150, 150 to 200, and 200 to 300 µm. Yang et al. [54] studied silicone rubber with 39 and 46.5 vol.% microspherical ZnO varistors with diameters ranging from 50 to 75, 75 to 100, 100 to 125, and 125 to 150 μm [54]. They also worked with two different morphologies: in composites of 46.5 vol.% ZnO, spherical particles were sieved into four groups with diameters ranging from 50 to 75, 75 to 100, 100 to 125, and 125 to 150 μm, and irregularly shaped particles were also sieved into four groups with dimensions ranging from 20 to 35, 35 to 50, 50 to 75, and 75 to 125 μm [52]. Finally, Yang et al. [56] studied the effect of grain size on the properties of microvaristors and their composites using three sintering temperatures, 1220 °C, 1120 °C, and 1020 °C for Z1, Z2, and Z3 fillers, respectively. The results of these studies are shown in Figure 10 and Figure 11 and Table 5.

Figure 10 and Table 5 show that the switching electric field decreased significantly with the increase in the filler size. α increases slightly from 14.96 to 16.08 [46], while it remained stable (~10) for composites with 39 vol.% ZnO [54] and increased to 17.5 for 46.5 vol.% ZnO with the largest size. No significant changes were observed in α in [52] with the increase in the filler size, but *E_b_* and α were higher for irregularly shaped fillers than those for spherical fillers. In addition, Figure 11 and Table 5 show that the switching electric field increased significantly with the decrease in grain size.

**Figure 12:**
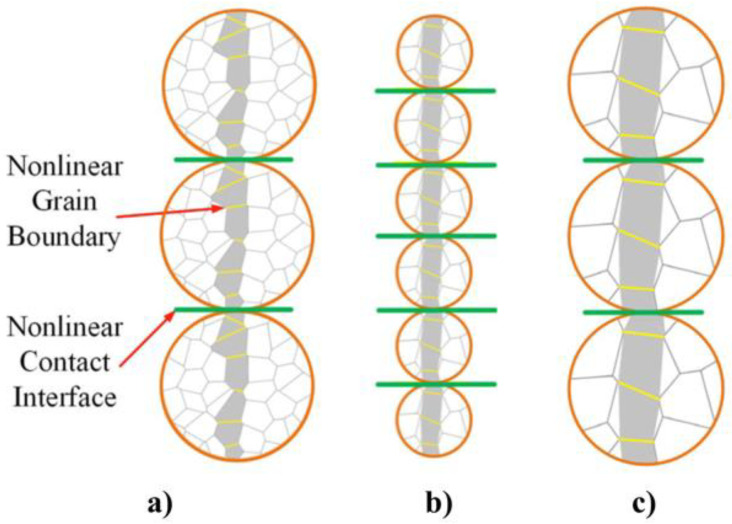
Schematic of conduction paths in nonlinear composites. (**a**) Large diameter particle with small grain size, (**b**) small diameter particle with small grain size, (**c**) large diameter particle with large grain size. © 2021 IEEE. Reprinted, with permission, from [56].

The nonlinear conduction of composites containing microvaristor fillers is largely determined by conduction paths formed by the fillers. When the filler concentration is below the percolation threshold, the composite behaves as an insulator. Conduction paths begin to develop above the percolation threshold, where the composite exhibits nonlinear properties [52]. At a given filler concentration, a larger filler size leads to fewer filler particles in the composite (Figure 12). For randomly distributed fillers, the probability of fewer and larger fillers forming a conduction path is higher than that of many smaller fillers. Then, more conduction paths lead to shorter main conduction paths, resulting in lower switching fields for composites with larger filler particles [52]. In addition, the volume resistivity of composites is related to the number and resistance of contacts [46]. For the same filler volume fraction, the number of contacts and contact resistance between the biggest particles are smaller than those between smaller particles (Figure 12a,b) [56]. For the composites with the larger filler size, there is lower contact resistance at each interface. When the lower electric field is applied, more charge carriers can be transported through the polymer. A conduction path is easier and quicker to be switched on. Therefore, composites with larger fillers present sooner nonlinear electrical properties [46]. The effect of the filler grain size on the switching field of composites is illustrated in Figure 12a,c. The circles represent contacting ZnO microvaristor fillers forming conduction paths in composites. Polygons within the circles represent the grains of ZnO. The current flows through more grain boundaries in a single conduction path when the grain size is smaller. As each grain boundary possesses a similar switching voltage (around 3 V), the higher the number of grain boundaries in the conduction path, the higher the switching field of the composites [56].

#### 4.1.3. Effect of Chemical Treatment

**Figure 13:**
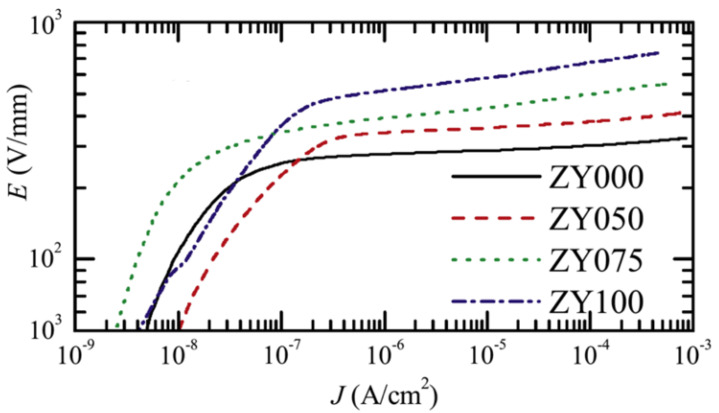
*J–E* characterization of ZnO varistors modified with Y_2_O_3_. Reprinted from [79], Copyright 2011, with permission from Elsevier.

Because the nonlinear properties of composites depend on the chemical structure of ZnO, it is possible to control *E_b_* and α by modifying the bulk properties of the ZnO filler. For example, He et al. [79] studied ZnO varistors doped with different Y_2_O_3_ concentrations. The compositions of ZnO varistors were (95.05-*x*) mol % ZnO, 0.70 mol % Bi_2_O_3_, 0.50 mol % MnO_2_, 1.00 mol % Co_2_O_3_, 0.50 mol % Cr_2_O_3_, 1.00 mol % Sb_2_O_3_, 1.25 mol % SiO_2_, and x mol % Y_2_O_3_, where x = 0, 0.50, 0.75, and 1.00, respectively [79]. When the Y_2_O_3_ concentration increased, the grain size in the fillers was 10.13, 7.22, 6.85 and 6.03 μm for 0, 0.50, 0.75, and 1.00 mol %, respectively [79], indicating grain growth inhibition with the introduction of Y_2_O_3_ [79]. The nonlinear behavior of the composites is presented in Figure 13 and Table 6.

Figure 13 and Table 6 show that the switching field of varistors increased and the nonlinear coefficient decreased as the Y_2_O_3_ concentration increased. The grain size was also modified by the addition of Y_2_O_3_. This modification explains the changes in the nonlinear properties of composites.

**Figure 14:**
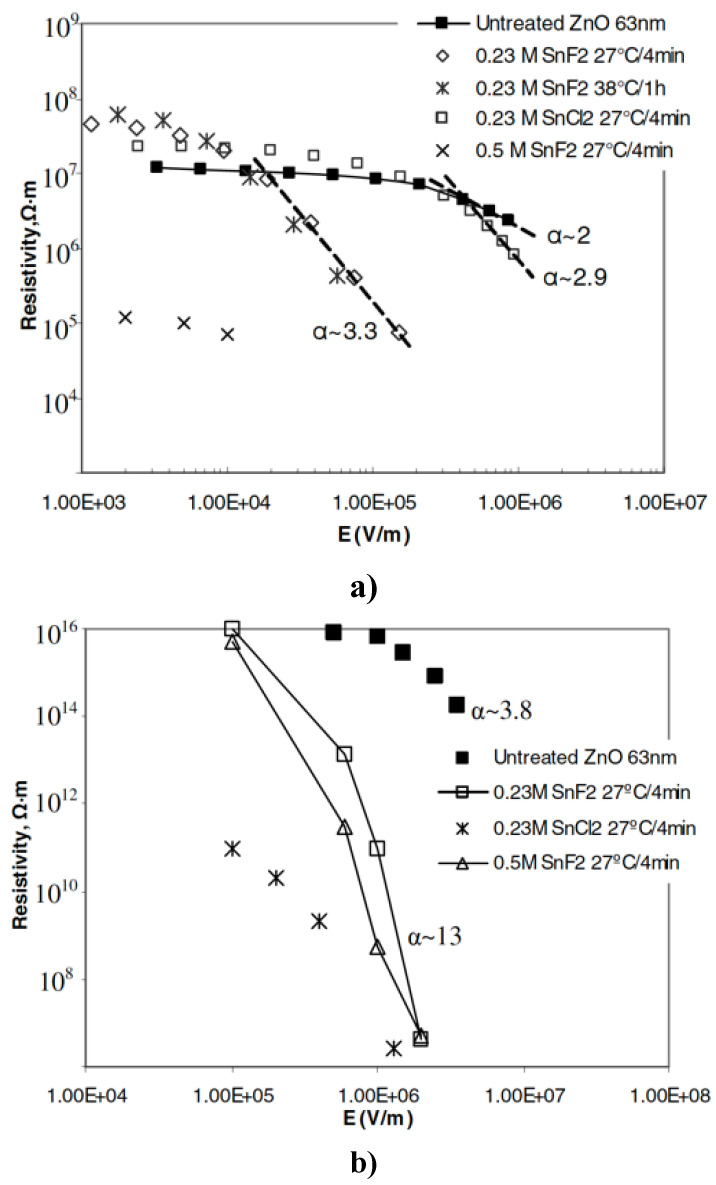
Resistivity versus electric field of (**a**) treated fillers and (**b**) 20 vol.% ZnO/EPDM nanocomposites. © 2021 IEEE. Reprinted, with permission, from [58].

Wang et al. [58] fabricated a composite with EPDM and 20 vol.% ZnO nanoparticles or 20 vol.% ZnO nanoparticles treated with aqueous SnX_2_ (X = F or Cl). The particles had a size of 63 nm. The electrical characterizations are presented in Figure 14.

As shown in Figure 14a, the treated fillers showed enhanced nonlinear conduction compared to pure ZnO [58]. With controlled treatments, the conductivity can remain constant at low field, and the onset of nonlinearity and the nonlinear coefficient can be adjusted through surface treatment [58]. For example, by increasing the concentration of SnF_2_ solution, the conductivity of fillers can be increased [58]. For composites, Figure 14b shows the resistivity as a function of the electric field [58]. The nonlinear coefficient and conductivity increase for composites with ZnO treatment. It can be deduced that the higher conductivity of SnO decreases the barrier for tunneling through the polymer matrix, and the higher nonlinearity in the composites could be due to the much larger conductivity of the treated powders at the fields of interest (10^5^ V/m) compared to untreated ZnO [58].

### 4.2. SiC-Based Composites

Another particle used for nonlinear applications is silicon carbide (SiC). As for ZnO fillers, the electrical properties of composites can vary based on SiC type, size, and concentration. In this section, the impacts of these factors are described.

#### 4.2.1. Effect of Filler Concentration

**Figure 15:**
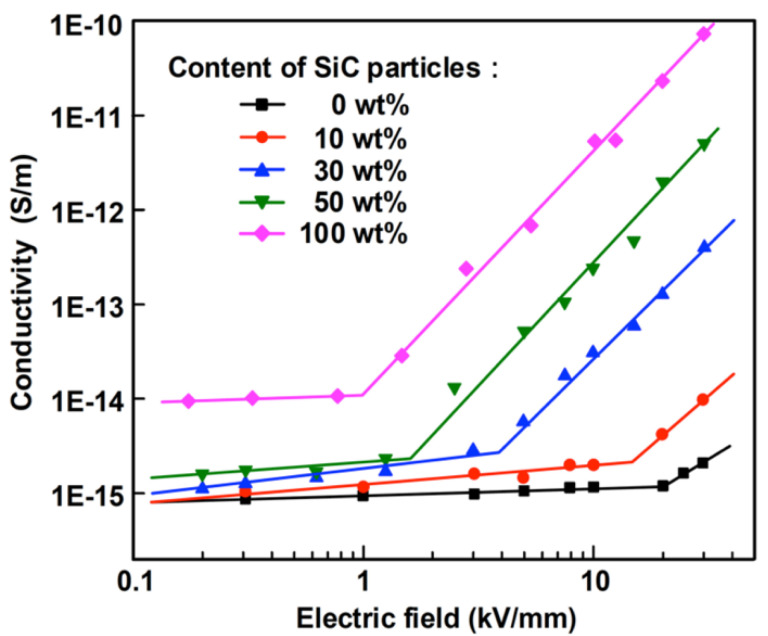
Relationship between conductivity and electric field for SiR/SiC composites. © 2021 IEEE. Reprinted, with permission, from [59].

The first factor described is the filler concentration. Du et al. [59,60] studied composites with silicone rubber (SiR) and SiC fillers. The fillers were spherical α-SiC hexagonal crystals with an average diameter of 0.45 μm. In the first work, the filler contents were 10, 30, 50, and 100 wt.%. In the second work, the composites included SiO_2_ (20 wt.%) with a diameter of 30 nm for structural reinforcement. Hexamethyldisilazane (5 wt.%) was introduced as a coupling agent to modify the SiO_2_ surface so that nanoparticles could distribute uniformly [60]. Additionally, structure control (1.3 wt.%) and vulcanizing (1.2 wt.%) agents were included. The SiC concentrations used were 10, 30, and 100 wt.% [60]. The results are presented in Figure 15 and Table 7, which show that SiR/SiC composites exhibited nonlinear conductivity as a function of the electric field when the concentration of SiC exceeded 30 wt.%. Their switching electric field decreased with increasing filler concentration. The nonlinear coefficient remained constant around 1. Slight nonlinearity was observed for neat SiR. At low electric fields, the materials possessed ohmic conductivity. However, for high-electric-field regions, the conduction showed a space-charge-limited current (SCLC) mechanism [59]. Based on the percolation theory, for composites with low filler content (below the percolation threshold), the mean distance between particles is so large that no conducting paths can be formed throughout the composites [59]. Further increase in the filler content and exceeding the percolation threshold lead to a decrease in the mean distance between the particles, resulting in the formation of conduction paths within the matrix [59]. The DC conductivity increased exponentially when the electric field exceeded the threshold field. The switching fields of the nonlinear conductivity were much lower than the inflection points of the SCLC effect due to the percolation theory [59]. Under low fields, SiR/SiC composites were in the ohmic conductivity region. In this region, the carriers meet difficulties to overcome the potential barrier at the interface of SiC particles. When the electric field increases and exceeds a certain threshold field, electric-field-assisted tunneling occurs between neighboring particles. In this case, a large number of carriers pass through the interface of particles, and the conductivity of SiR/SiC composites rises macroscopically, resulting in nonlinear conductivity [59].

**Figure 16:**
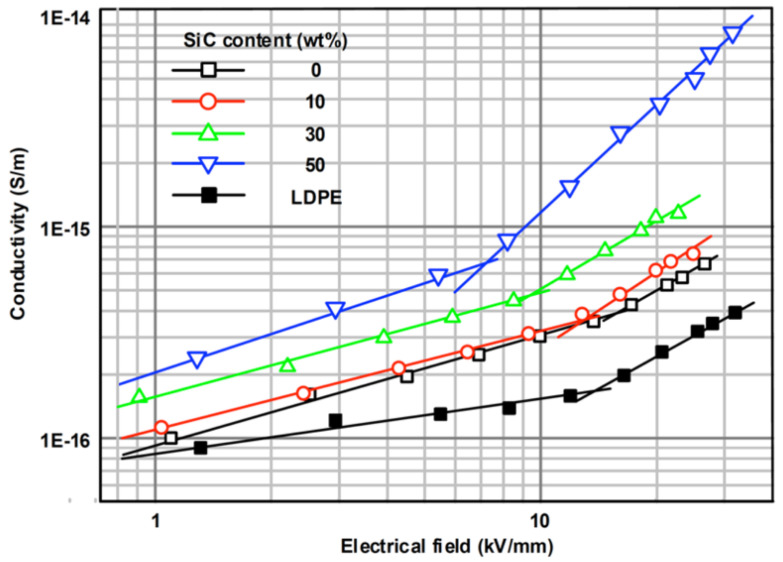
Relationship between conductivity and electric field for EPDM/SiC composites. © 2021 IEEE. Reprinted, with permission, from Ref. [61].

Li et al. [61] studied the effect of the filler concentration in composites of ethylene propylene diene terpolymer and SiC (β-crystal) as fillers with a diameter of 0.5 μm. Their results are presented in Figure 16 and Table 7.

As depicted in Figure 16 and Table 7, the switching field decreases with the increase in SiC concentration [61]. The nonlinear conductivity of EPDM/SiC composites was explained by the tunneling effect through the interface barriers and hopping conduction. Under low field strength, very few carriers can get through the interface barriers by thermal excitation and participate in the hopping conduction in the polymer, leading to a relatively low bulk conductivity. When the field strength exceeds a certain threshold, the tunneling effect occurs. SiC particles introduce a large number of charge carriers into the composite bulk, thereby promoting the migration of carriers under local stress. The carrier mobility increases when the filler concentration increases because the average distance between SiC particles decreases, giving rise to a whole or partial conductivity network in the matrix [61].

#### 4.2.2. Effect of Filler Shape and Size

**Figure 17:**
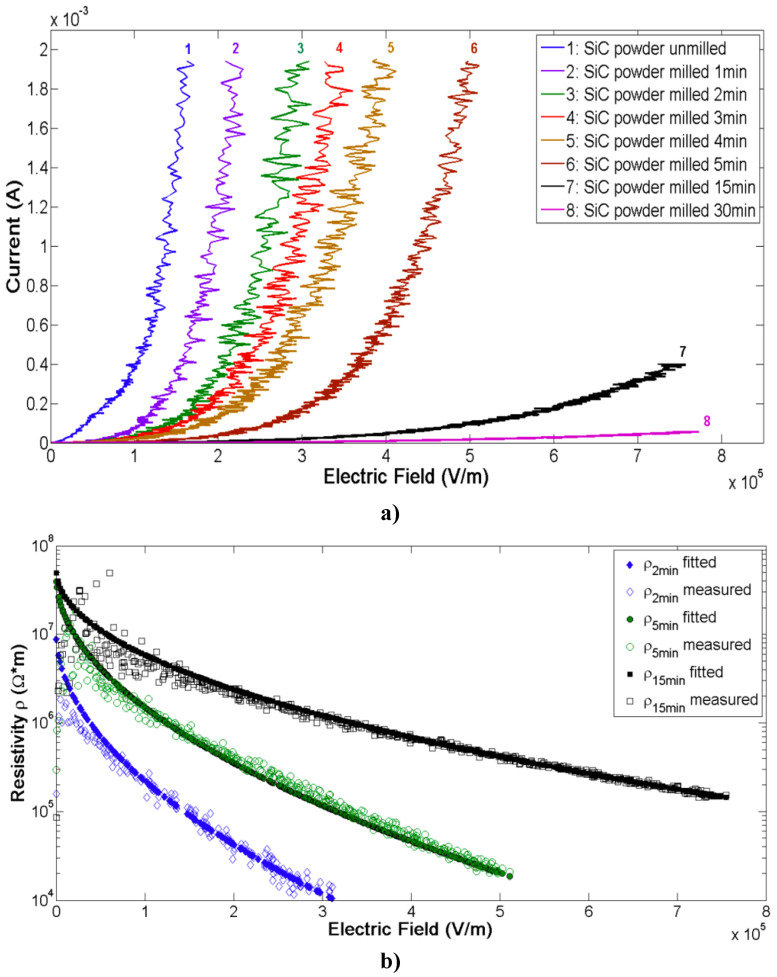
Electrical characterization of SiC powders for different milling times. (**a**) Current vs. electric field, (**b**) resistivity vs. electric field. © 2021 IEEE. Reprinted, with permission, from [63].

The effects of particle size on the nonlinear properties of SiC-based composites were studied. Vanga-Bouanga et al. [63] used commercially available black SiC powder with a purity of 99.9% and an average particle size of 50.6 µm. To alter the distribution of SiC particle sizes, a ball milling process was conducted using an impact grinder for up to 30 min with 15-mm diameter balls in a crucible. The electrical characterization of composites with modified SiC fillers is shown in Figure 17.

**Figure 18:**
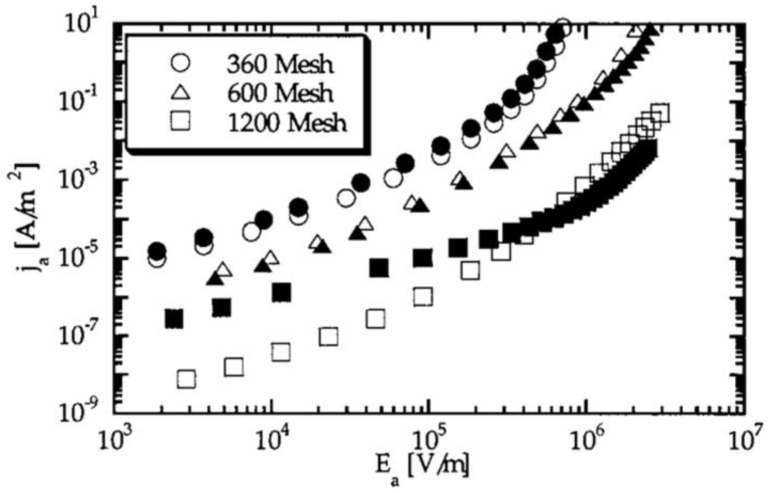
Current density as a function of the average applied electric field. Green, n-type SiC powders of different grain sizes at room temperature. Open/closed symbols define dry/moist samples. Reprinted from ref. [64] Copyright 2001 AIP Publishing.

The SiC particle size decreased with the ball milling time (1, 2, 3, 4, 5, 15, and 30 min), and the obtained sizes were 33.17, 23.36, 16.33, 13.22, 7.32, 3.28, and 2.06 µm, respectively. As shown in Figure 17a, particles had nonlinear conduction, and this behavior strongly depended on the SiC particle size: the current decreased when the filler size decreased. Figure 17b shows that the resistivity of the filler decreased when the milling time increased. Mårtensson et al. [64] studied the grain size effect in SiC fillers using 360, 600, and 1200 mesh corresponding to median grain diameters of 22.8, 9.3, and 3.0 µm, respectively. The current density as a function of the average applied electric field is presented in Figure 18.

**Figure 19:**
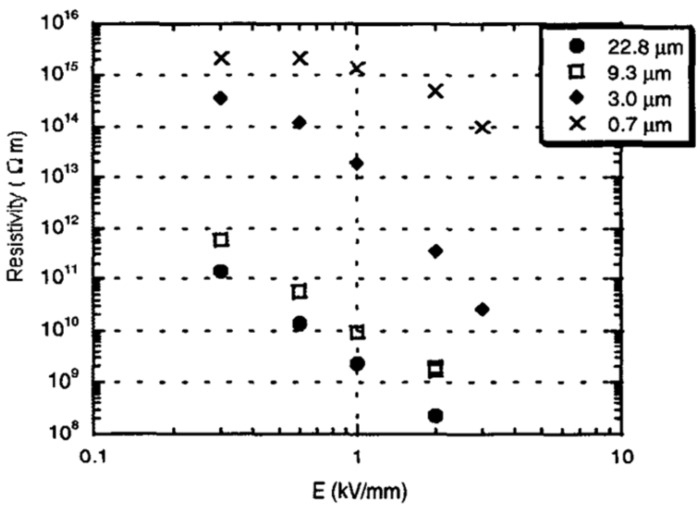
Resistivity as a function of electric field for composites involving SiC particles of various sizes. © 2021 IEEE. Reprinted, with permission, from [62].

The grain size remarkably affects the properties of fillers (Figure 18). When the grain size decreased, the current density also decreased, and nonlinearity was observed for all sizes. Moreover, Onneby et al. [62] studied the impact of the filler size in SiC-based composite materials. They worked with a matrix of EPDM with 40 vol.% SiC particles with average grain sizes of 22.8, 9.3, 3.0, and 0.7 μm. The effect of the filler size on the resistivity of the composites is presented in Figure 19.

All the composites exhibited nonlinear electrical characteristics; however, the switching field increased when the grain size decreased. In the composites with particle sizes of 22.8 and 9.3 μm, the materials showed nonlinearity even at low electric fields. The resistivity of the composites also decreased when the filler size increased. The decrease in resistivity results from the fewer grain-to-grain contacts needed to obtain a conducting path and the increased voltage across each contact.

In general, the behavior is the same as that obtained for ZnO: if the filler size increases, the probability to form conducting paths increases, and the contact resistance decreases; thus, the switching field of composites decreases.

#### 4.2.3. Effect of Chemical Treatment

Mårtensson et al. [64] studied two SiC fillers (9.3 μm) with different chemical compositions: n-type green SiC (with 5 × 10^24^/m^3^ N and 4–6 × 10^24^/m^3^ Al) and p-type black SiC (with ≈10^26^/m^3^ Al).

**Figure 20:**
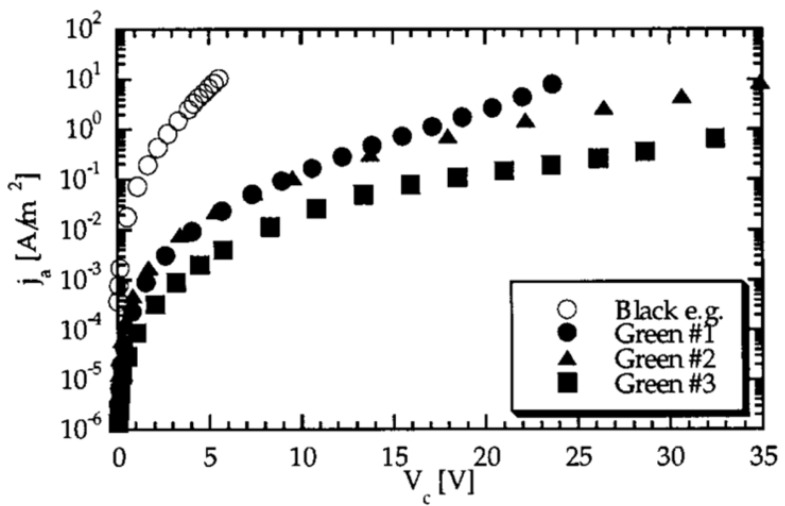
Current density as a function of voltage for black and green SiC. Reprinted with permission from ref. [64] Copyright 2001 AIP Publishing.

**Figure 21:**
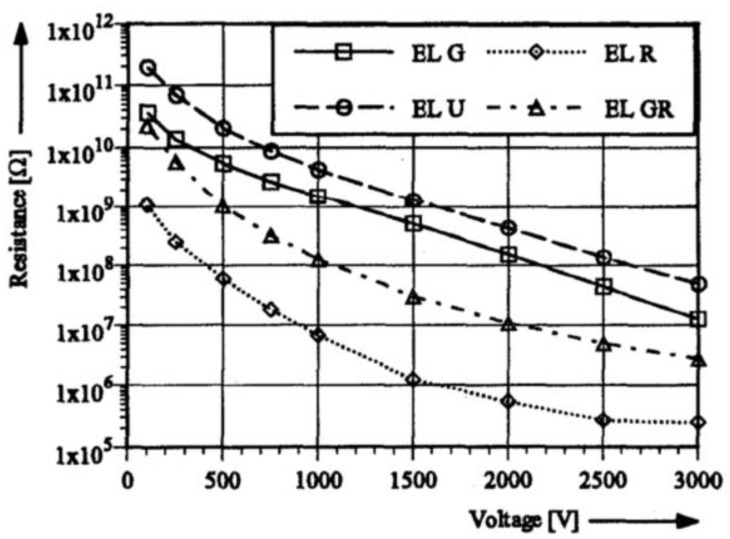
Influence of doping in SiC/epoxy composites. © 2021 IEEE. Reprinted, with permission, from [65].

Figure 20 shows a sample of black SiC and three samples of green SiC. The black SiC had a higher conductivity and nonlinearity. The significant difference between the two doped grains confirms that the electrical conductivity of composites can be controlled by filler composition. Gärtner et al. [65] studied SiC-based composites without doping and with p-type (Al) and n-type doping. The characteristics of the fillers are presented in Table 2. The composites were fabricated with an epoxy resin base matrix, and the electrical resistance was studied as a function of voltage. All samples were based on the same SiC grain size (F280/F100 = 25/75) and bulk factor (82%). EL G and EL GR were made of n-type SiC, EL U, undoped, and EL R, p-type SiC. The electrical results are presented in Figure 21.

The materials fabricated with undoped fillers had the highest electrical resistance. When the fillers were n-type, the electrical resistance of composites decreased compared with that of the undoped one. Finally, the composite with the lowest resistance was Al-doped p-type SiC [65]. The filler composition influenced the final properties of the composites.

In conclusion, the nonlinear properties of composites with SiC can be controlled through filler characteristics (doping, size, and concentration) in the polymer matrix. These parameters are very important to obtain specific properties for intended applications.

### 4.3. Composites Based on Carbon Allotropes

**Figure 22:**
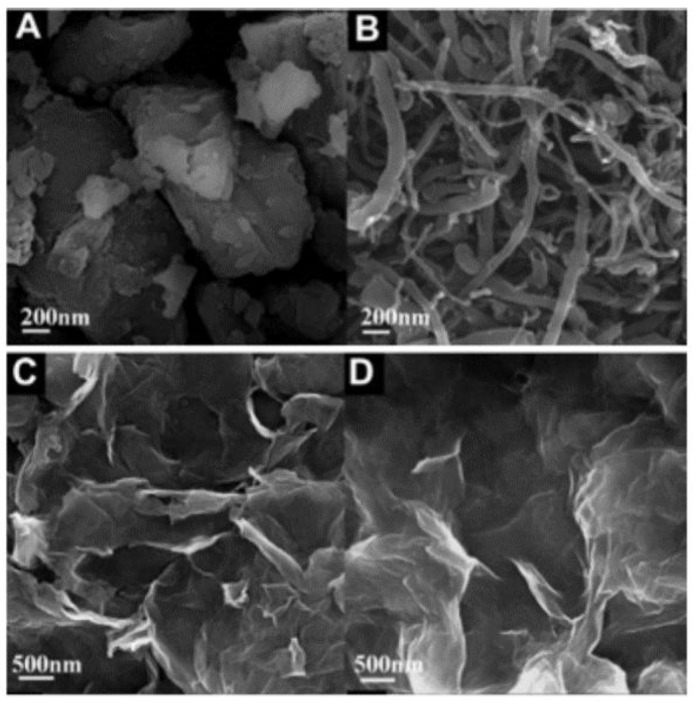
Typical FESEM images of (**A**) fullerenes (C_60_), (**B**) CNTs, (**C**) graphene oxide, (**D**) solvent-exfoliated graphene. Reprinted from [81], Copyright 2013, with permission from Elsevier.

Carbon allotropes are usually used as fillers in various applications when higher electrical conductivity is required. They are classified according to their dimensionality (zero-, one-, two-, and three-dimensional particles). Fullerenes are zero-dimensional, carbon nanotubes (CNTs) are one-dimensional, monolayer graphene are two-dimensional, and carbon black (CB), graphite (GP), and graphene nanoplatelets (GNPs) are three-dimensional [80]. In Figure 22 [81], the typical FESEM images of four carbon allotropes are presented.

In addition, the structures of the allotropes are different. Ideally, graphite comprises infinite layers of sp^2^-hybridized carbon atoms. Within a layer (graphene sheet), each C atom bonds to three others, forming a planar array of fused hexagons [82]. The unhybridized 2p_z_ orbital that accommodates the fourth electron forms a delocalized orbital of π symmetry that further stabilizes the in-plane bonds [82]. The weaker van der Waals force keeps the layers stacked with a distance of 3.354 Å. Graphite is a good electrical conductor in the plane directions (due to the delocalized π band) and a poor electrical conductor in the stacking direction (due to the van der Waals force between the layers). Fullerenes and SWCNTs form when the dangling bonds at the edges of a real (finite) graphene layer are connected, changing the sp^2^ hybridization state. Depending on the rolling of the graphene sheet, metallic or semiconducting CNTs can be obtained. Nanotubes can also have different diameters, be open-ended or closed-ended (resembling a very long fullerene), and single-walled or multi-walled (a concentric array of carbon cylinders ~3.4 Å apart is observed), and either of these parameters affects the properties of materials [82]. When these fillers are used in the fabrication of composites, their aspect ratios affect filler dispersions within the polymer, and particles with a distinct aspect ratio such as CNTs build a stable and interconnected network above the percolation threshold, enabling fast and reproducible changes in the properties [80]. In contrast, particles with arbitrary aspect ratios such as CB, GP, and GNPs do not build such an interconnected network, and the properties can be changed by only the direct contact of particles.

Other structures based on carbon are amorphous carbons, such as CB and carbon fibers. These materials feature rough planar layers of mostly sp^2^-hybridized carbon atoms, but they lack long-range crystallinity, especially in the stacking direction. They contain a significant fraction of sp^3^ carbon atoms, which often cross-link with neighboring layers, forming an overall structure that comprises amorphous and more graphitic regions [82].

#### 4.3.1. Composites Based on Amorphous Carbon

Materials fabricated with CB possess nonlinear electrical conductivity, such as PE [66], natural rubber vulcanizates [83,84], and HDPE [67]. For instance, Nakamura et al. [66] fabricated composites using CB with a surface area of 19 m^2^/g and a size of 90 nm at different concentrations. Three different behaviors were observed [66]: For low filler concentration, the current showed ohmic behavior because the distance between fillers was very large, preventing the formation of conducting paths [66]. When the filler concentration increased (intermediate concentration), the current showed a dramatic increase with the increased applied field. In this region, the distance between particles was small enough to form conducting paths induced by tunneling conduction. Finally, for high filler concentration, the ohmic dependency of the current versus the applied field appeared due to the direct contact between fillers, and the influence of new conducting paths was negligible due to the tunneling effect. Further, Tang et al. reported the nonlinear behavior in composites based on HDPE with different concentrations of carbon black [67]. The electrical behavior of these composites exhibited three regions. In region 1 (low electric field), as the voltage increased, the resistivity remained almost constant at a value equal to that of an insulator up to a certain voltage (switching voltage). In region 2, the resistivity largely decreased as the voltage increased (nonlinear behavior). In region 3, the resistivity remained almost constant with the increased voltage. The switching voltage strongly depended on the CB ratio. The conductivity of this composite depended on electron tunneling, which is the dominant transport process in HDPE/CB composites [67]. When the CB concentration increased, the distance between two conducting CB aggregates decreased, and the tunneling conduction appeared, resulting in a decrease in the switching voltage.

**Figure 23:**
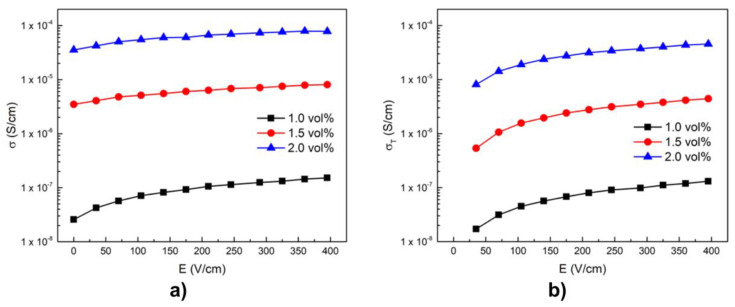
(**a**) σ and (**b**) $\sigma_{T}$ as a function of electrical field for CNF/HDPE composites of various filler concentrations at room temperature. Data extracted from Ref. [68].

He et al. reported the nonlinear behavior in composites based on HDPE with carbon nanofibers (CNFs) fabricated by melt compounding [68]. The CNFs had a diameter in the range 100–200 nm. The effect of the electric field on the conductivity of composites at room temperature is shown in Figure 23. $\sigma_{0}$ is the conductivity when the electric field is removed, and $\sigma_{T}\left(E\right)$ represents the tunneling conductivity induced by the electric field.

The conductivity of the composites increased with the increasing electric field, as shown in Figure 23. The whole conductivity can thus be written as $\sigma \left(E\right)=\sigma_{0}+\sigma_{T}\left(E\right)$. Figure 23b shows that the tunneling effect increases when the filler concentration increases. In general, the electrical conductivity of carbon-based conducting polymer composites originates from two mechanisms: ohmic and non-ohmic conduction [68]. The former originates from the direct contact of conducting fillers at and above the percolation threshold. The latter occurs through the barrier tunneling effect between conducting fillers separated by a thin polymer layer. In other words, tunneling conduction takes place when the distance between the filler is close, generally less than 10 nm.

#### 4.3.2. Composites Based on Carbon Nanotubes

Carbon nanotubes, discovered by Iijima [85], played an important role in the field of polymer composites [86]. Carbon nanotubes are divided into two types: SWCNTs and MWCNTs [85,86]. SWCNTs form cylinders made up of graphene sheets with well-defined atomic structures and high length-to-diameter ratios, and they are regarded as one-dimensional molecules due to their chemical stability. CNTs have three types based upon their atomic arrangement: armchair, zigzag, and chiral structures [86]. They can be either metallic or semiconducting depending on this structure [85]. MWCNTs resemble a collection of concentric SWCNTs having a 0.34-nm interlayer distance [85,86]. Their properties depend on their diameter, size, and morphology [86].

**Figure 24:**
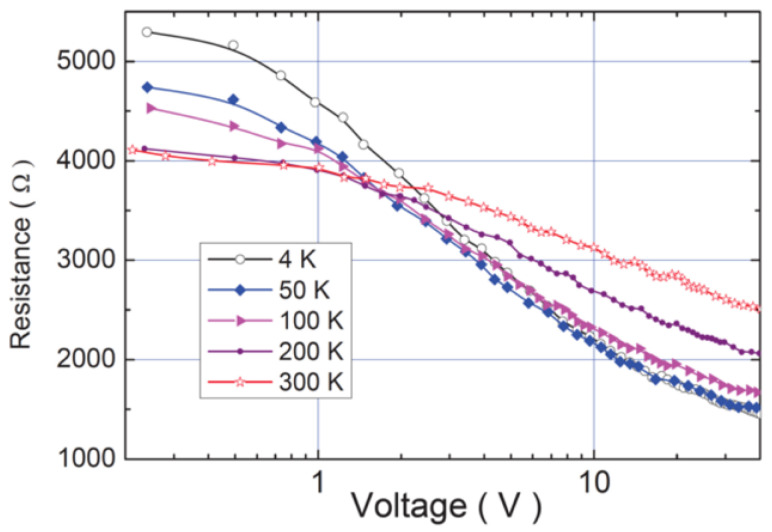
Dependence of SWCNT resistance versus applied voltage at different temperatures. © 2021 IEEE. Reprinted, with permission, from Ref. [70].

De Pablo et al. [69] studied the nonlinearity in SWCNTs using single nanotubes with diameters of about 2 nm and different lengths. They reported that the resistance of CNTs increased when the nanotube length increased and that the electrical current (resistance) was a nonlinear function of the voltage. Subačius et al. [70] also studied the nonlinear behavior of SWCNTs. CNTs produced by electric-arc-discharge using nickel-yttrium catalyst with diameters between 1.2 and 1.5 nm were stuck in bundles with an average length of 3.5 μm and diameters in the range 3–7 nm. Thin SWCNT films with a thickness of about 100 nm were prepared by spraying the SWCNT suspension (0.04 g/L) on a hot glass substrate with previously prepared Au contacts [70]. Their results are presented in Figure 24 for different temperatures.

The electrical resistance decreased when the voltage and temperature of the composites increased. The nonlinearity depended on sample resistance upon the applied voltage, and strong electric field induced nonlinear effects in the electrical conductivity of SWCNT films [70]. The increase in the electric conductivity with the increasing applied electric field strength was attributed to the tunneling between the conducting paths of metallic SWCNT. At low electric fields, the conductivity of SWCNT layers is dominated by the tunneling of carriers through the insulating gaps. Thermal fluctuation facilitates the tunneling probability, and the semiconducting resistance is observed. Electric field modifies the potential barriers between the conducting regions in such a way that the effective barrier width and height are reduced; consequently, sample resistance decreased [70].

Bliznyuk et al. [71] reported nonlinearity in polyurethane-based (PU) composites using SWCNTs as fillers. They prepared the materials by mixing the polymers with the CNTs (0.25–2 wt.%) in a solution of 1-methyl-2-pyrrolidone (NMP) by sonication. The total concentration of nanotubes was 1 wt.%. The PU/CNT composites showed a nonlinear I–V relationship.

On the other hand, Liu and Fan investigated the electrical conductivity of MWCNT networks in flexible polydimethylsiloxane (PDMS) rubber as a function of applied voltage [72]. CNTs were grown by CVD and were mostly MWCNTs with diameters around 10 nm and lengths around 10 μm. The PDMS nanocomposites were prepared by ultrasonic dispersion with CNT concentrations ranging between 0.1–3 wt.%. For the different concentrations of MWCNTs, the I–V curves were nonlinear. The electrical resistance of the samples largely varied from that at low voltages. The nonlinear mechanism was related to the material system including both MWCNTs and the PDMS matrix. As in the case of SWCNTs, they followed a fluctuation-induced tunneling mechanism in which the thermally activated voltage fluctuation across the insulating gaps played an important role in determining the temperature- and field-dependent conductivity [72].

#### 4.3.3. Composites Based on Graphite and Modified Graphite

**Figure 25:**
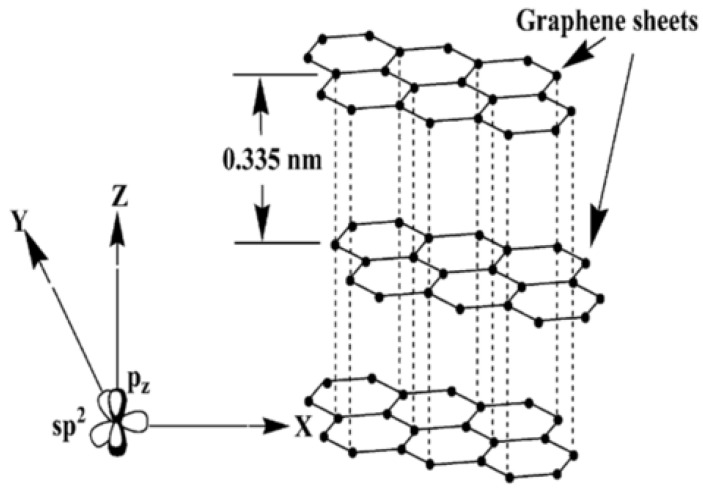
Layered graphite structure showing sp^2^-hybridized carbon atoms tightly bonded in hexagonal rings. Reprinted from [87], Copyright 2011, with permission from Elsevier.

The graphite crystal lattice consists of stacks of parallel two-dimensional (2D) graphene sheets (sp^2^ graphene layer or graphene sheet) with hybridized carbon atoms tightly bonded in hexagonal rings, as shown in Figure 25 [75,76,87]. Since the 2p_z_ orbitals of carbon atoms can overlap most effectively when they are parallel (out-of-plane *π* bond), the graphene sheet has the lowest energy when it is completely flat [87]. Due to the difference between the in-plane and out-of-plane bonding of carbon atoms, graphite is anisotropic. The *π* orbital is distributed over the entire graphene sheet, making it thermally and electrically conductive [86,87]. The adjacent graphene sheets in graphite are separated from each other by 0.335 nm, the adjacent graphene sheets are held together by weak van der Waals forces; thus, graphene sheets can easily slide with respect to each other.

In its bulk state, graphite exists as a layered material. For the efficient utilization of graphite as a filler in a polymer composite, its layers must be separated and dispersed throughout the polymeric matrix. The basic unit obtained by the exfoliation of the natural flaky graphite is the graphite nanoplatelet (GnP) having a platelet thickness varying from less than 0.34 to 100 nm, and its theoretical surface area is 2630–2965 m^2^/g [86,87]. In its natural form, no reactive ion groups exist on graphene layers, and as a result, it is impossible to intercalate monomers into graphite galleries by ion-exchange reactions [87]. GnPs are mainly obtained from sulfuric acid-intercalated graphite and GO. Broadly, three treatment methods are adopted for graphite modification, which forms GO, graphite intercalated compounds (GICs), and expanded graphite (EG).

GO, also known as graphite oxide, graphitic oxide, or graphitic acid, is usually prepared by the treatment of graphite flakes with oxidizing agents so that polar groups are introduced on the graphite surface, thereby widening the interlayer spacing of graphene planes. Due to the modification of the structure, GO is electrically insulating [87].

GICs are formed by the insertion of the atomic or molecular layers of different chemical species between the layers of the graphite host lattice [87]. The number of graphite layers between the intercalated layers is known as the stage, and the most important and characteristic ordering property of GICs is the staging phenomenon.

EG is another form of modified graphite. When the intercalated graphite (most commonly graphite bisulfate, although any GIC can be used) is heated beyond a critical temperature or exposed to microwave radiation, a large expansion of graphite flakes occurs along the c-axis, forming vermicular or worm-like low-density accordions with high temperature resistance called EG [87]. EG is composed of stacks of nanosheets that may vary from 100 to 400 nm. EG exhibits a layered structure similar to layered silicates and has a good affinity to organic compounds and polymers.

The final graphite form is graphene, which can be prepared by five methods: The first involves the CVD of graphite monolayer on transition metal surfaces [87]. The second is the micromechanical exfoliation of graphite and involves the peeling of graphene from graphite using “Scotch” tape. The tape is then dipped in acetone to release graphene, which is subsequently captured on a silicon wafer with a SiO_2_ layer on top. The third involves the epitaxial growth of graphene on electrically insulating substrates, such as silicon carbide. However, the second and third methods are unsuitable for the large-scale preparation of graphene for the fabrication of polymer nanocomposites. Bulk quantities of graphene and chemically modified graphene are obtained from GO and GICs. Due to the presence of hydroxyl and epoxide groups on graphitic basal planes, and carboxyl and carbonyl groups on the edges of layers, GO is water-dispersible. Thus, it is possible to apply sonication in aqueous media to get colloidal suspensions of single GO layers. Afterward, it is possible to chemically reduce GO in the colloidal suspension using reducing agents, such as hydrazine or hydrazine derivatives, to convert electrically insulating GO back to conducting graphene [87]. The last method to obtain bulk quantities of graphene is the thermal reduction of GO [75,76,87]. This technique involves rapidly heating GO in an inert (argon or nitrogen) environment to produce thermally reduced expanded graphene oxide (TRGO), which is a black powder of very low bulk density. The heating is responsible for the exfoliation of graphene sheets. TRGO is also known as functionalized graphene sheets (FGSs) and has a wrinkled sheet structure due to the epoxy groups, forming chains across the graphene surface. Due to their wrinkled nature, FGSs do not collapse back to GO but remain highly agglomerated [87].

Due to their characteristics, graphite and modified graphite have been used for the production of composites with nonlinear conductivity. Hereafter, some works reported in the literature are presented.

**Figure 26:**
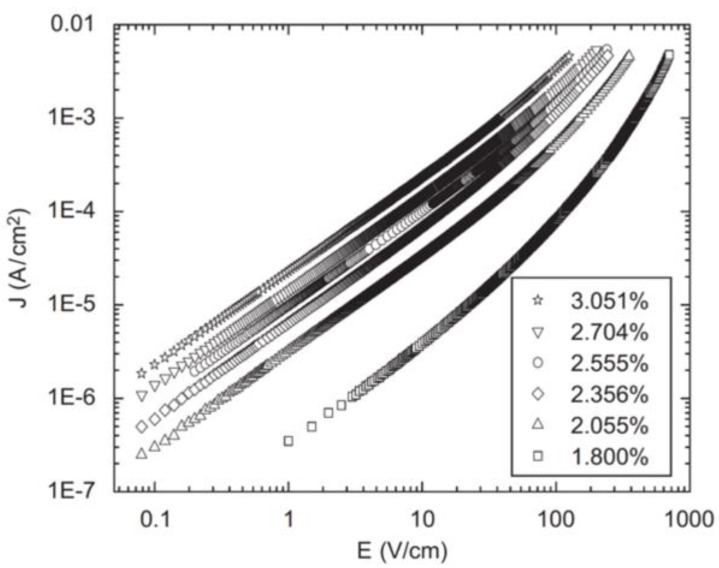
Current density (*J*) vs. electric field (*E*) for GNs/epoxy composites with different filler contents. Reprinted from [73], Copyright 2007, with permission from Elsevier.

Lin et al. [73] reported an epoxy-based composite using graphite nanosheets (GNs) as fillers. GNs had thicknesses between 30 and 80 nm and diameters between 5 and 20 μm with an average thickness of about 50 nm (the number of sheets in the platelets is 150) and a mean diameter of roughly 12 μm. They investigated the nonlinear conduction of GNs/EP composites above the percolation threshold by the action of variable DC electrical field. Their results are presented in Figure 26 and Table 8.

For GN/epoxy composites the relation between I and V was nonlinear. As can be observed in Figure 26 and Table 8, the current density increased with increasing GN concentrations. Current–field characteristics had a linear relationship at low electrical fields for each curve, and they had a nonlinear relationship at high fields. They reported a percolation threshold (P_c_) around 1.35 vol.%.

It is highlighted that the closer the specimen to the percolation threshold, the smaller the linear response regime (switching field). The increase in nonlinearity near P_c_ could be due to the conducting network formed by very few conducting paths. Insulating epoxy resin existed inside the clusters of GNs because of the physical interaction between the GNs and matrix. When the gaps between the clusters were small, intercluster and intracluster tunneling or hopping under sufficiently high fields across insulating gaps provided new conducting pathways, giving rise to supplementary nonlinearity.

**Figure 27:**
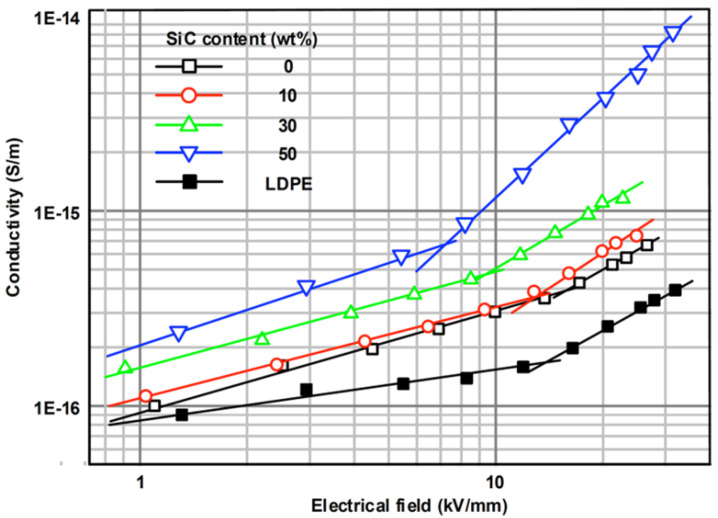
Conductivity (σ) vs. electric field (*E*) for GnP/LDPE with different concentrations. Reprinted from [74].

Gaska et al. studied composites of LDPE filled with different concentrations of GnPs in the form of films using a pre-coating technique and single-screw melt extrusion [74]. The GnPs had a surface area of 120–160 m^2^/g, average diameter of 25 μm, thickness of 6–8 nm, and density of 2.2 g/cm^3^. The composites were fabricated using two types of screws during the extrusion: a compression screw (CS, compression ratio 2:1) and a mixing screw (MS, compression ratio 5:1). In both cases, the filler concentrations were 0, 1, and 5 wt.%. The dependence of conductivity as a function of the electric field is presented in Figure 27.

Figure 27 shows that samples with 1 and 5 wt.% GnPs showed lower DC conductivity compared to pure LDPE. They explained that GnP particles act as charge-trapping sites, reducing the transport of electric charges through the material. This effect was the most significant at a lower filler concentration (1 wt.%) and a relatively low electric field. For all composites, a nonlinear behavior was observed with a switching field of about 20 kV/mm. A clear crossover effect was also reported, where the conductivities of the filled nanocomposites exceeded that of pure LDPE at high field strength. This effect was the strongest in LDPE composites containing 5 wt.% GnPs. They also observed a tendency for the conduction to saturate at fields about 40 kV/mm for pure LDPE and above this level for the nanocomposites.

**Figure 28:**
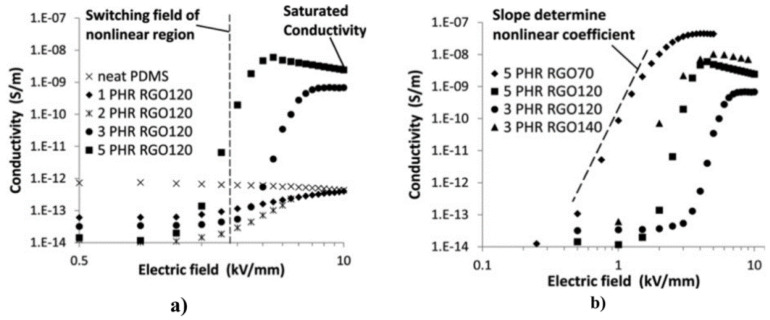
Conductivity vs. field strength for (**a**) composites with different filler contents of GO reduced at 110 °C and (**b**) composites with GO reduced at different temperatures. Reprinted with permission from [88]. Copyright 2012 John Wiley and Sons.

Moreover, Wang et al. worked with SiR composites using GO as fillers [75,88]. The average lateral dimension of GO was about 500 nm, and the average thickness was 1.1 nm, indicating that it was mostly monolayer GO. They studied the effect of the thermal reduction of GO in the properties of composites using different temperatures between 70 °C and 160 °C for 12 h. They also studied the effect of the filler concentration using volume fractions between 1 and 5 PHR (parts per hundred parts of resin). Their results are presented in Figure 28.

**Figure 29:**
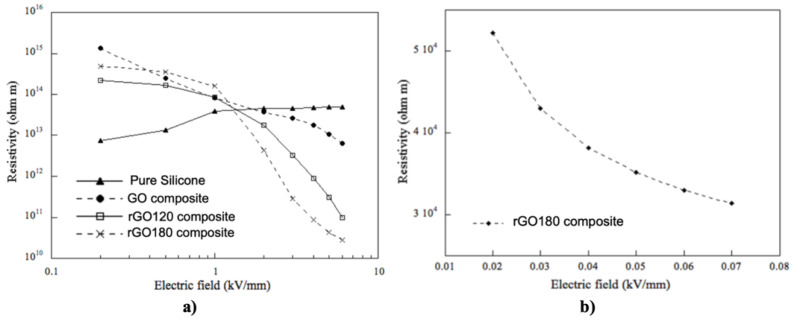
DC resistivity of (**a**) SiR (triangles) and composites based on GO (circles), rGO120 (squares), rGO180 (cross), and (**b**) rGO220 (diamonds) as a function of electric field at 23 °C. © 2021 IEEE. Reprinted, with permission, from [76].

Figure 28a shows the conductivity of composites filled with GO reduced at 120 °C as a function of the electric field [88]. At low field strength (below 1 kV/mm), all composites exhibited a lower conductivity than that of the neat PDMS. At higher fields, the conductivity of the composites increased dramatically, while the electronic current dominated the ionic current. This nonlinear conductivity can be described by the switching field and the slope in the nonlinear region, as shown in Figure 28. In the nonlinear region of GO-filled composites, α had a value of 16, which is a relatively large value in field-grading applications. For samples with filler concentrations of 1 PHR and 2 PHR, the high field conductivity of the composites was similar to that of neat PDMS because the GO content was below the percolation threshold. For filler concentrations of 3 PHR and 5 PHR, the conductivity increased by several orders of magnitude at high fields due to the intrinsic field-dependent conductivity of GO. Abundant surface groups led to a disrupted sp^2^ structure and acted as energy barriers for charge transport along the carbon network. Electrons were blocked by those energy barriers at low electric fields and became capable of tunneling through them only at high electric fields. Thus, the electronic conduction along the GO network was facilitated at elevated fields and became the major contributor to the total current in percolated samples. Figure 28b shows the ability to tailor the properties of composites by altering the GO oxidation state. Each energy barrier, from either the GO surface groups or the contact between GO platelets, possesses a characteristic voltage above which electrons can tunnel through. The total switching voltage of the composites should be the sum of those characteristic voltages. Therefore, adjusting the oxidation state of GO can affect energy barriers from surface groups and eventually change the total switching voltage. By increasing the reduction temperature from 120 °C to 140 °C (3 PHR–RGO120 vs. 3 PHR–RGO140), the switching field shifted from 4 kV/mm down to 2 kV/mm because of a reduced number of oxidized surface groups and energy barriers. Li et al. [76] also studied thermally reduced GO using SiR as a matrix. GO was heated at different temperatures between 120 and 220 °C. The composites were fabricated with 3 wt.% fillers. The electrical characterization is presented in Figure 29.

At low electric fields (≤1 kV/mm), all composites exhibited higher resistivity than that of the pristine SiR. An increase in the resistivity by up to two orders of magnitude was observed. The higher resistivity in the GO-filled composites compared to that of the pristine SiR was due to a tortuous path of ion movements created by the GO network. At higher electric fields, the resistivity of reduced graphene oxide (rGO)-based composites decreased by four orders of magnitude. The electronic current dominated the ionic current due to the intrinsic field-dependent conductivity of rGO. The composites showed a nonlinear resistivity, and the switching field of nonlinearity started close to 1 kV/mm. The pristine SiR exhibited insulating properties over the entire electric field range studied. The composite with GO showed a very high resistivity (1.3 × 10^15^ Ωm). It decreased only moderately to 6.3 × 10^12^ Ωm at 6 kV/mm and remained insulating even at the highest electric field. For composites containing rGO120, the remaining functional groups on the basal plane behaved as deep traps [76]. At lower electric fields, electrons were trapped in those groups, whereas at high electric fields, they tunneled through the network. Composites containing rGO180 were more conductive at electric fields greater than 1 kV/mm [76]. Due to the highly reduced state of rGO220, these composites became much more conductive than others (Figure 29b). The resistivity was between 3.0–5.5 × 10^4^ Ωm at low electric fields in the range 0.02–0.07 kV/mm. The significant increase in the electrical conductivity of rGO220 composites was due to the removal of the majority of oxidized groups in GO sheets. In conclusion, when GO is used in the fabrication of composites, the nonlinearity can be tuned by adjusting the reduction degree of GO.

## 5. Polymer Composites with Multiple Particle Types

**Figure 30:**
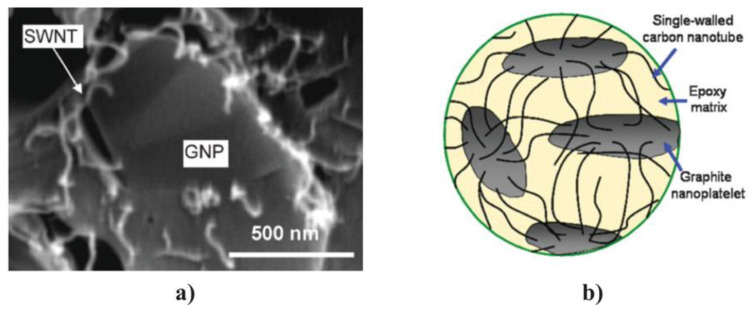
GNP/SWCNT/epoxy composite: (**a**) SEM image and (**b**) schematic representation of GNP-SWCNT network. SWCNTs bridge adjacent graphite nanoplatelets, and SWCNT ends are extended along nanoplatelet surfaces. Reprinted with permission from [89]. Copyright 2008 John Wiley and Sons.

As discussed in Section 4, the properties of composites depend on filler properties. Many fillers can be used to obtain properties different than that obtained with one filler type. The idea of using two or more fillers is to improve composite performance by combining their advantages [89]. For instance, this technique was used to modify the electrical and optical properties of chitosan-ZnO composites by dispersing GO and ZnO in the chitosan matrix [90]. Also, the electrical conductivity of composites using GO-ZnO particle combination was ~4.131 × 10^6^ S/cm, which is higher than those using only GO, where the conductivity was limited to ~0.231 × 10^6^ S/cm. Further, the dielectric response of composite materials was investigated based on ZnO/BaTiO_3_/EP [91]. They were prepared in different filler concentrations, and the dielectric response was studied by broadband dielectric spectroscopy. The dielectric permittivity and loss of ZnO/BaTiO_3_/EP increased with ceramic filler content and diminished rapidly with frequency. The functionality of the composite systems is related to the abrupt variation of the real part of permittivity near the characteristic Curie temperature of BaTiO_3_ and the polarization of ZnO particles. The simultaneous presence of both effects in hybrid composites generates bifunctional response. Ouyang et al. [92] studies the optical properties of graphene/ZnO/poly(methyl methacrylate) (PMMA) organic glass composites. They reported that the nonlinear optical coefficient of the graphene/ZnO/PMMA organic glass was approximately 5.6 and 7.8 times larger than that of graphene/PMMA and ZnO/PMMA organic glasses, respectively. The enhancement in the nonlinear optical properties was related to the positive synergistic effects between graphene and ZnO. Yu et al. [89] and Lee et al. [93] worked on the thermal properties of composites. Yu group worked with an epoxy resin as a matrix and purified SWCNTs and graphite nanoplatelets comprised of few graphene layers (n~4) as fillers (10 wt.%). The results showed a pronounced maximum thermal conductivity with GNP:SWNT filler ratio of 3:1 (7.5 wt.% GNPs and 2.5 wt.% SWNTs in epoxy); thus, the hybrid fillers demonstrated a strong synergistic effect and superseded the performances of individual SWCNT and GNP fillers [89]. The electrical conductivity also showed a nonmonotonic behavior as a function of the GNP fraction with a minimum reached for a GNP:SWNT filler ratio of 1:3. Figure 30 shows an SEM image of an epoxy composite with a filler content (GNP:SWCNT) of 3:1 (7.5 wt.% GNPs and 2.5 wt.% SWNTs), which corresponds to the maximum observed in the thermal conductivity. The images show complex nanostructures with multiple SWCNTs bridging adjacent GNPs.

Pradhan et al. [53] studied multifunctional composites with different characteristics, such as hydrophobicity and high tracking performance, fire resistance and/or fire extinguishing capability, favorable electrical stress-grading properties, and high thermal conductivity. For that purpose, they developed materials with different properties using silicon rubber as the matrix and ZnO and other functional fillers. Six functionally graded materials (FGMs) were developed, as shown in Table 9. 

**Figure 31:**
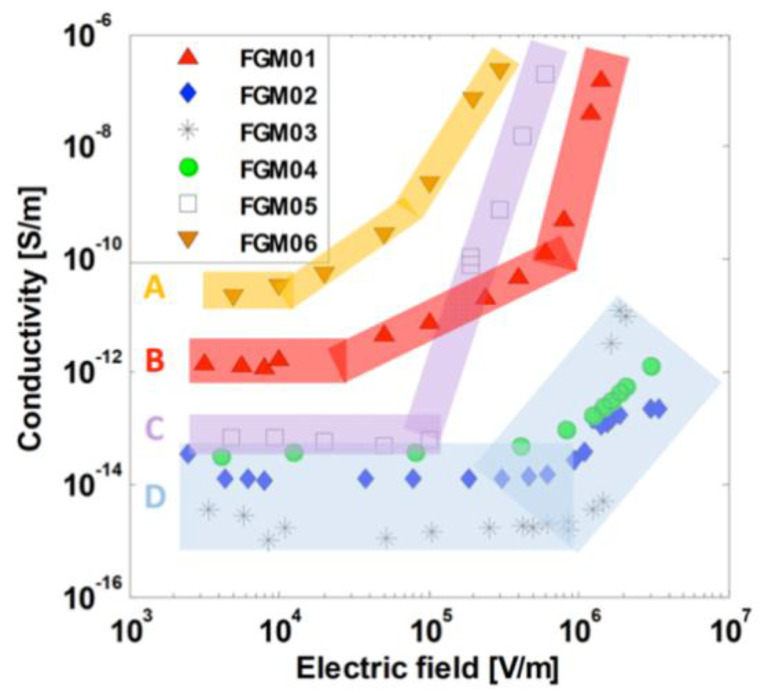
Log–log plot of DC conductivity at 30 °C vs. electric field of the field-grading materials with different compositions. © 2021 IEEE. Reprinted, with permission, from Ref. [53].

The diameter of ZnO microvaristors was less than 90 μm with an average length of 60.5 μm [53]. The electrical characterization is presented in Figure 31 and Table 9 where it can be observed that FGMs can be divided into four classes. Class A materials have the highest low-field conductivity (10^−11^ S/m) and *E_b_* around 1 × 10^4^ V/m, often considered for corona protection on stator bars in rotating machines. The conductivity and *E_b_* progressively decreases and increases, respectively, to reach class D, which has a low-field conductivity between 10^−15^ and 10^−14^ S/m and *E_b_* around 1 × 10^6^ V/m [53]. Additionally, permittivity and dielectric loss both degrade (i.e., slight increase) reasonably for uses in HV applications, while the thermal conductivity of the composites presents a relatively interesting increase.

For field-grading properties, Mårtensson et al. [94] fabricated composite materials using an EPDM matrix filled with both SiC and CB. The sizes of SiC and CB particles were 10 and 1 μm, respectively. The composites were formed by 17.5 vol.% SiC and 11.7 or 13.1 vol.% CB. The electrical characterizations showed a nonlinear behavior. An increase in the amount of CB gave a higher conductivity at low electric fields. When the concentration of CB increased, the switching field and the nonlinear exponent of the composites decreased. The SiC grains were mostly isolated and were not in contact with each other. CB grains served as links between the semiconducting SiC particles. When the amount of CB increased, the system percolated better. Thus, the conductivity at low field, determined by the matrix plus percolated chains of CB alone, increased. CB grains may short-circuit certain SiC contacts, while a lower nonlinearity threshold field and nonlinearity exponent could be expected.

Finally, Hu et al. reported the characterization of composites fabricated with an epoxy resin and different fillers (nano-SiC, nano-ZnO, micro-ZnO) [95]. The mass fractions of inorganic fillers were 1, 3, and 5 wt.%. The composition and nomenclature of each composite are presented in Table 10.

**Figure 32:**
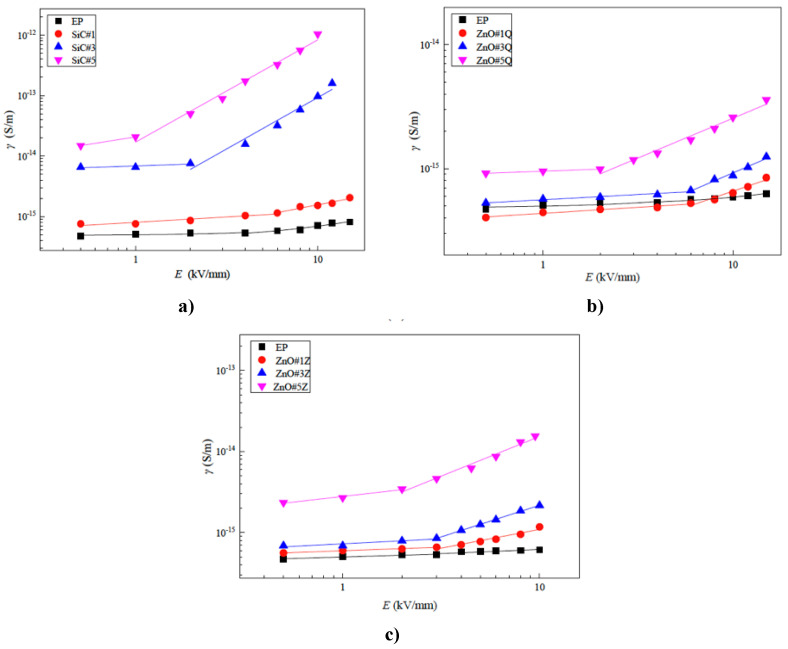
Conductivity as a function of electric field for composites with different fillers. (**a**) nano-SiC/EP composites, (**b**) nano-ZnO/EP composites, and (**c**) micro-ZnO/EP composites. Reprinted from [95].

The electrical characterization of these composites with individual fillers is presented in Figure 32.

**Figure 33:**
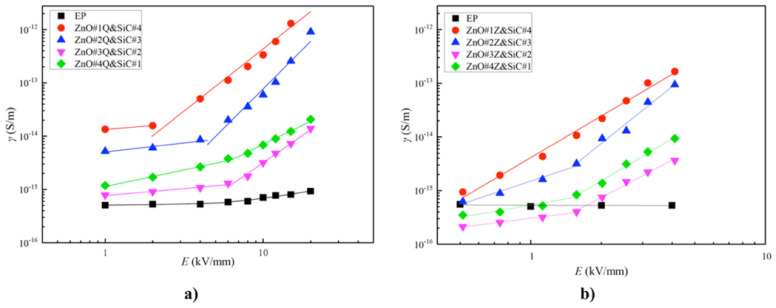
Conductivity–electric field characteristic curves of epoxy-based composite materials with different fractions of inorganic fillers: (**a**) nano-ZnO/SiC/EP nanocomposites and (**b**) micro-ZnO/SiC/EP micro/nanocomposites. Reprinted from [95].

As described in the previous sections, the switching field intensity decreases and the conductivity increases with increasing the filler concentration [95]. The nonlinear coefficients of composite materials are significantly higher than that of undoped epoxy resin, and the nonlinear coefficients increase with increasing filler concentrations. For the same filler concentration, the nonlinear coefficients of nano-SiC/EP composites are the highest. Micro-ZnO/SiC/EP micro/nanocomposites and nano-ZnO/SiC/EP nanocomposites were also prepared. In both cases, the total mass fraction of inorganic fillers was 5 wt.%. The ratios of ZnO to SiC were 1:4, 2:3, 3:2, and 4:1. The characteristic curves of conductivity–electric field are presented in Figure 33, and the switching field and nonlinear coefficients of conductivity are presented in Table 10.

Figure 33 shows that the electrical conductivities of both composites increased, and their threshold field strength decreased with increasing SiC concentration. Therefore, SiC determined the conductance characteristics of ZnO/SiC/EP composites. In Table 10, the nonlinear coefficients of composites involving using two inorganic filler types were superior to those of single-filler composites. At a ZnO:SiC ratio of 2:3, the nonlinear coefficients of nano-ZnO/SiC/EP and micro-ZnO/SiC/EP reached maximum values of 2.96 and 3.50, respectively. Therefore, the micro-ZnO inorganic filler had a greater influence on the nonlinear coefficients. Compared to the composites of 5 wt.% SiC/EP, micro-ZnO/EP, and nano-ZnO/EP, the nonlinear coefficient of 5 wt.% 3:2 micro-ZnO/SiC/EP composites was greater by a factor of 0.82, 2.48, and 5.01, respectively [95]. These results show that working with a combination of different fillers helps to tune the final electrical properties of composites and obtain different nonlinear conduction properties, depending on application specifications.

## 6. Anisotropic and Nonlinear Polymer Composites

Forming conducting paths with fillers is important to obtain nonlinear polymer composites. The position and orientation of filler particles inside the matrix impact the final properties of composites, particularly when particles are non-spherical. Techniques or processes to handle the position and formation of conducting paths inside a polymer matrix and their impact on the final properties of composites were studied. For example, an original design to control the properties of composites is to develop FGMs, which involve materials whose composition and/or microstructure vary continuously in one or several directions [96,97]. Several preparation techniques were developed for these composites, depending upon their physical state and specific applications [98,99]. Constituents can be mixed in solid, liquid, or gaseous phases prepared through solid, liquid, and gaseous phase processing, respectively.

In gaseous phase processing, constituents are boiled to their vapor state and then condensed after thorough mixing to form a solid FGM with the required microstructure and properties. The commonly used gas-based methods to fabricate FGMs are CVD, chemical vapor infiltration, physical vapor deposition, ion plating, plasma spraying, and ion mixing [98,99].

Liquid-phase processing can be divided into different methods: centrifugal, combustion, casting, and deposition methods. In the centrifugal method, constituents are mixed in a liquid or liquid–solid state and poured into a casting cylinder. Due to differences in their densities, they form graded layers of different densities when the cylinder rotates. In the combustion method, constituents react exothermically and produce heat energy, which melts them. The commonly used combustion techniques are reactive combustion forging, rolling, pressing, extrusion, and casting. In the casting method, constituents in a powder form are mixed thoroughly in a solution of solvent and plasticizer; then, it is cast or solidified to the required geometry. In the deposition method, a solution of powder constituents is prepared. Then, the deposition of these materials is triggered by an electric, chemical, or laser field using electrodeposition, chemical solution deposition, or laser deposition. Commonly used liquid-phase processing methods include sedimentation, electrochemical gradation, and directional solidification [98,99].

The solid phase processing of FGMs generally involves the processing of constituent materials in a solid state. The constituent materials may be processed in powder form. Powder metallurgy is assumed as the simplest method for the preparation of FGMs. In this method, mixtures of powder constituents are kept in a die, compacted, and then sintered to get the final product. The commonly used methods are 3D printing [100,101,102,103,104], solid-state foaming, and solid freeform laminate/stack processing [98,99].

Functional grading methods used in the literature for the fabrication of polymer composites and micro- and nanofillers are liquid-phase processing. Extrusion [105,106], infiltration [107,108], electrical deposition methods [109,110,111,112,113,114], magnetophoretic deposition [115], centrifugal method [96,116,117], and gravity-assisted casting [97] have been reported to produce FGMs, as discussed below.

Badini [105] reported the fabrication of SiC whiskers/aluminum 6061 composites by extrusion using two different ratios of extrusion. The difference in the ratio generates different degrees of alignment and fracture in whiskers. The degree of orientation of the matrix and reinforcement crystals increases as the diameter of the extruded material decreases. The mechanical properties of the materials showed anisotropy: its compressive strength in the longitudinal direction was considerably higher than that in the transverse direction [105]. Qu et al. [106] also worked on extrusion and reported the effect of filler orientation on the electrical conductivity of composites. The matrix material was PMMA, and the fillers were carbon fibers with a diameter of 7 μm, initial length of 6 mm, density of 1.79 g/cm^3^, and specific resistance of 1.7 × 10^−3^ Ω/cm. The composites were extruded through a capillary rheometer, utilizing either 1-mm- or 3-mm-diameter extrusion dies, resulting in cylindrical composite filaments of two different diameters. They reported that the average CF orientation became more aligned with the extrusion flow when the diameter of the extrusion dies decreased [106]. This orientation impacted the percolation thresholds of composites, which were 20.0 ± 2.5 vol.% and 32.0 ± 5.9 vol.% for 3-mm and 1-mm filaments, respectively. The oriented CFs in the composites shifted the percolation threshold to a higher value. Below 32 vol.% fibers, the conductivity of 3-mm composites was higher than 1-mm composites, but the opposite was observed above 32 vol.% [106]. Thus, the orientation of particles influenced the percolation threshold and produced anisotropy in the final properties of the composites [107,108,118].

**Figure 34:**
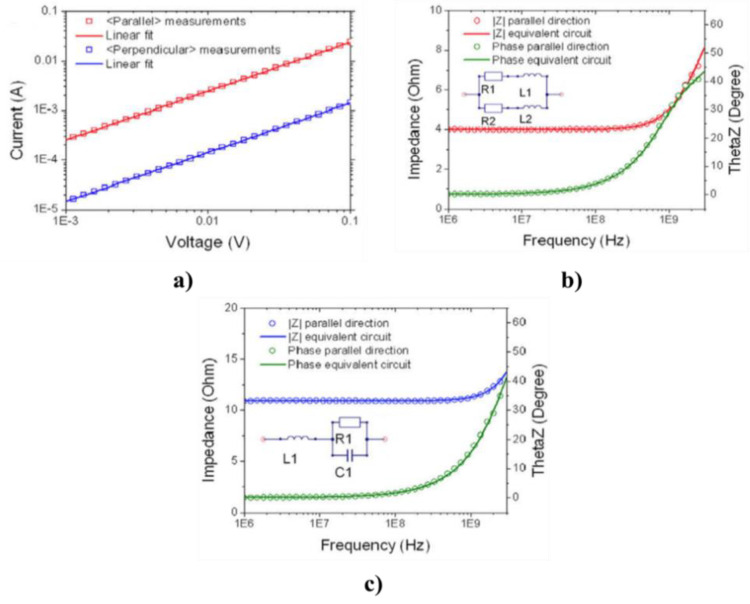
Electrical characterizations of MWCNT/EP composites. (**a**) Current–voltage data for composites, (**b**) impedance and phase vs. frequency data (circles) with the corresponding equivalent circuit model (lines) for the parallel direction, and (**c**) impedance and phase vs. frequency data (circles) with the corresponding equivalent circuit model (lines) for the perpendicular direction. Reprinted from [107], Copyright 2010, with permission from Elsevier.

Dombovari et al. [107] reported anisotropy in the electrical conductivity of aligned MWCNT/epoxy composites. The composites were fabricated by infiltrating the films of CNTs with a commercial epoxy [107]. The MWCNT films were grown in a laboratory-made catalytic CVD chamber with an area of 10 × 10 mm^2^ and length of ~2 mm. Then, the films were punctured with a plastic tube. Subsequently, an epoxy resin was poured onto the punctured film. After hardening (12 h), the sample was removed from the punch, cut into smaller pieces, and polished to a size of ~2.0 × 2.0 × 1.4 mm^3^ [107]. The electrical characterizations in DC and AC are presented in Figure 34.

As presented in Figure 34a, in DC the current in the parallel direction was higher than the current in the perpendicular direction. Therefore, in the direction parallel to the fiber orientation, the conductivity was higher than that in the perpendicular direction. The comparison between parallel and perpendicular directions in AC (Figure 34b,c) shows that the impedance was lower in the direction parallel to the filler orientation [107].

**Figure 35:**
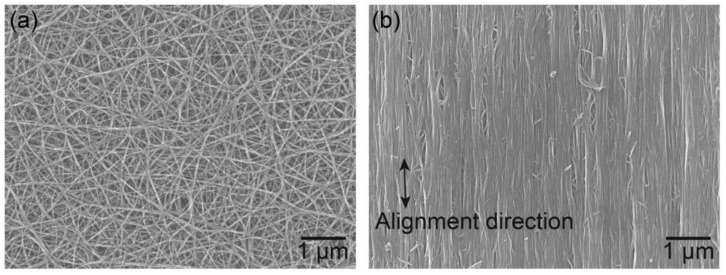
SEM images of (**a**) random and (**b**) aligned CNT sheets. Reprinted from [108], Copyright 2016, with permission from Elsevier.

Further, Yang et al. [108] worked on aligned carbon nanotube sheets reinforced with silicon carbonitride prepared by the infiltration and pyrolysis of liquid polysilazane into mechanically stretched MWCNTs (Figure 35). The diameter and length of MWCNTs in random CNT sheets were 6–8 nm and 1 mm, respectively. The composites were fabricated with a high volume fraction of CNTs up to 60 vol.% following a four-step process: (1) aligning CNT sheets, (2) infiltrating CNT sheets with polysilazane (CNT/PSZ), (3) exposure to high-temperature pyrolysis to generate CNT-reinforced silicon carbonitride nanocomposites (CNT/SiCN1), and (4) additional infiltration and pyrolysis to generate denser CNTs reinforced with silicon carbonitride (CNT/SiCN2).

**Figure 36:**
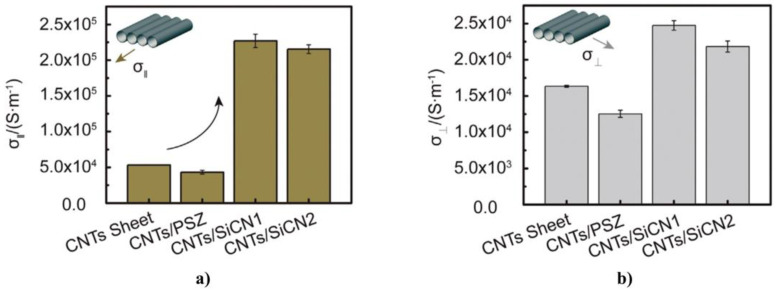
Electrical conductivity of aligned CNT nanocomposites: (**a**) parallel to the CNT alignment direction and (**b**) normal to the CNT alignment direction. Reprinted from [108], Copyright 2016, with permission from Elsevier.

The electrical conductivity of these composite materials was probed at each step, and the results in the parallel and perpendicular directions are presented in Figure 36.

During all the process stages, the electrical conductivity of CNT nanocomposites was higher in the direction parallel to the filler orientation. Therefore, it is possible to control the properties of the compound by controlling the manufacturing process and applying mechanical methods to produce a filler orientation. This orientation produces anisotropic properties with higher conduction in the parallel direction to the filler orientation.

**Figure 37:**
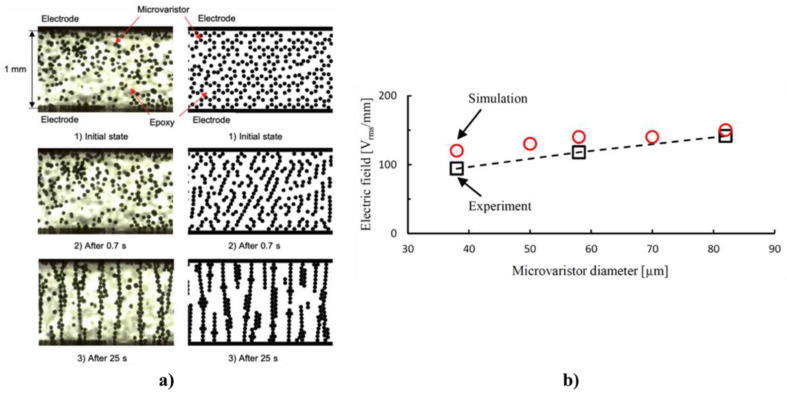
Comparison between experimental and simulated microvaristor deposition: (**a**) movement of fillers, (**b**) minimum electric field to form chains. © 2021 IEEE. Reprinted, with permission, from [110].

Nardi [115] et al. reported that a suspension of Fe_3_O_4_@TiO_2_ nanoparticles in an epoxy matrix induced the magnetophoretic motion of particles and high-aspect-ratio particle alignment under an external magnetic field. The combination of these two effects produces graded nanocomposites with permittivity gradients. Moreover, under an electric field between a pair of parallel-plate electrodes, particles may align to form a chain-like network in the direction of the electric field. This mechanism of self-aligning fillers in polymers, assisted by (di)electrophoresis, offers a great opportunity to create multiscale structures [109]. Komesu et al. [110] reported the application of an AC electric field (60 Hz) of 450 V_rms_/mm in a mixture of 20 wt.% microvaristors (average diameter of 58 μm) and epoxy resin poured between two parallel electrodes (Figure 37).

**Figure 38:**
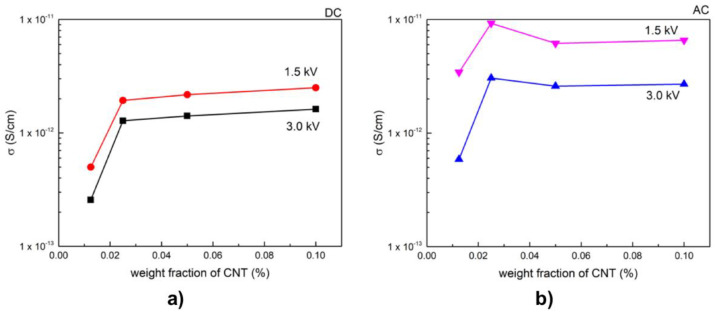
Electrical conductivities of polycarbonate/CNT composites as a function of nanotube weight fraction; (**a**) DC, (**b**) AC. Data extracted from [111].

They reported that when an AC electric field was applied during the curing process of the resin, microvaristors were aligned colinearly to the electric field. The formation of the chains was homogeneous so that the nonlinear electrical conductivity could be obtained anisotropically between the electrodes in the epoxy resin by forming the chains of microvaristors. The formation of filler chains was also modeled to obtain particle positions, and it was very similar to the experimental results [119]. The effect of applying an electric field has also been reported on the alignment and orientation of different particles, including CNTs [111,112,120], carbon fibers [109,113,114], and graphene nanoplatelets [114]. Larijani et al. [111] reported the effect of aligned carbon nanotubes under a DC and AC on the electrical conductivity behavior of polycarbonate nanocomposites. The CNTs were MWCNTs (diameter, 10–20 nm; length, 5–15 μm) with a purity higher than 95%. A mixture of CNTs, polycarbonate, and a solvent was poured on glass dishes, an AC and DC (V = 1.5–3 kV) were applied between two electrodes with a gap distance of 100 mm, and the solvent was left to evaporate slowly [111]. They reported that the application of the AC and magnetic field in this system led to the formation of continuous networks, and the applied DC only prevented the agglomeration of CNTs. They also reported the electrical conductivity of different tailored composites (Figure 38).

**Figure 39:**
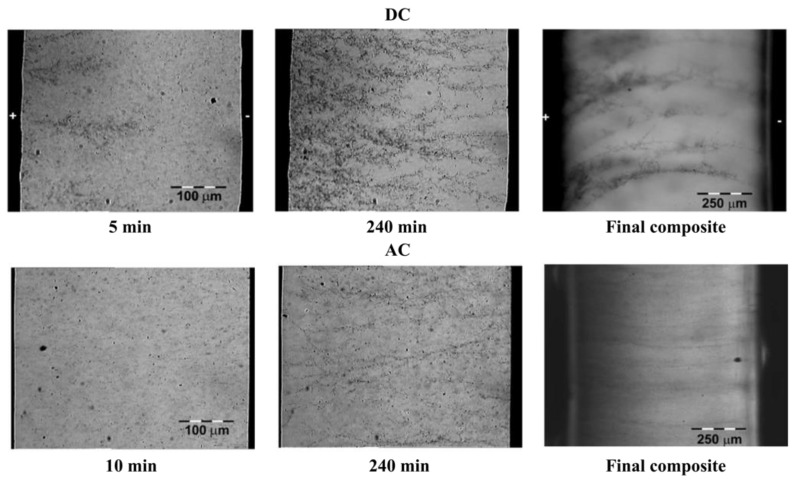
Transmission optical micrographs of epoxy composites containing 0.01 wt.% MWCNTs during curing at 80 °C using a DC and AC of 100 V/cm. Reprinted from [112], Copyright 2005, with permission from Elsevier.

The conductivities of polycarbonate/CNT composites where the particles were manipulated with an AC voltage were higher than those of the composites oriented by the DC voltage. This behavior is due to the formation of a chain network along the parallel direction of the electric field. When the electric field increases, the conductivity of the composites also increases due to the field-assisted chain construction that creates more and/or longer CNT chains in the interelectrode gap spacing. Martin et al. [112] also studied the orientation and alignment of CNTs in a liquid polymer using AC and DC. They reported MWCNTs (diameter: ~50 nm, length: 43 ± 3 μm) dispersed in an epoxy system based on bisphenol-A resin and an amine hardener. AC and DC were used during the curing of EP/CNT nanocomposites to align conductive nanotube networks between the electrodes. The EP/CNT nanocomposites were fabricated using different field strengths (DC and 1 kHz AC fields of 50, 100, and 200 V/cm) for a low CNT content (0.01 wt.%). The transmission optical micrographs are presented in Figure 39.

**Figure 40:**
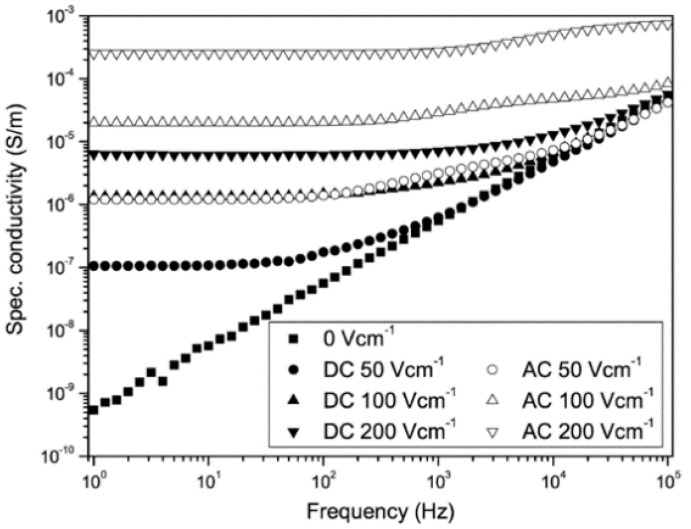
AC conductivity as a function of frequency for fully processed bulk epoxy nanocomposites containing 0.01 wt.% MWCNTs exposed to AC or DC during curing. Reprinted from [112], Copyright 2005, with permission from Elsevier.

As shown in Figure 39, the structured network formed in AC was more uniform and aligned compared to those obtained in DC. The influence of the CNT alignment saturation was studied using AC impedance spectroscopy under a low AC voltage magnitude (1 V_rms_) and frequencies ranging from 1 Hz to 10^5^ Hz. The results are presented in Figure 40.

In Figure 40, the conductivities of EP/CNT composites increased with the strength of the applied field. The reference (without any electric field application) showed a typical dielectric behavior, indicated by the frequency-dependent conductivity increase with a slope of unity when plotted in the double-logarithmic scale. On the contrary, all the other samples exposed to AC and DC showed frequency-independent conductivity, i.e., a DC horizontal plateau of more than 10^−7^ S/m at low frequencies. This constant conductivity was maintained up to a specific switching frequency, above which a transition to a dielectric behavior was observed. Furthermore, the AC led to significantly higher conductivity values than the DC of similar strength, by as much as an order of magnitude, reflecting the more homogeneous carbon nanotube network structure.

**Figure 41:**
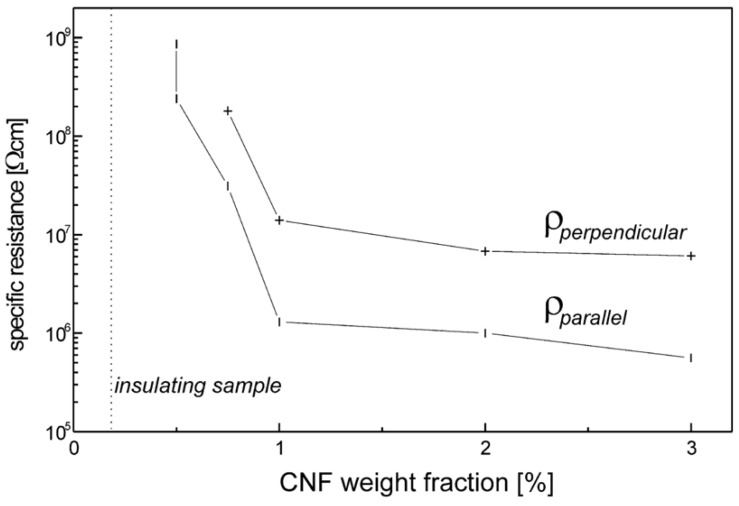
Resistivity as a function of CNF weight fraction in cured composites for directions parallel and perpendicular to the electric field. Reprinted from [113], Copyright 2003, with permission from Elsevier.

Ladani et al. [109] studied the alignment of CNFs in an epoxy resin during curing using an AC. The applied voltage was 60 V at 10 kHz to generate an AC electric field of 30 V/mm. During the curing of epoxy resin, CNFs were simultaneously observed showing rotation and alignment in the applied electric field direction to form a chain-like structure. Upon curing, the epoxy nanocomposites contained aligned CNFs. The effect of CNF alignment on the electrical conductivity of epoxy nanocomposites was measured for different filler contents (wt.%). The results for the AC and DC conductivities of randomly oriented and aligned CNFs composites were reported. In AC characterization, the conductivity increased when the filler content increased. Moreover, composites with aligned fillers exhibited higher AC conductivity. The same behavior was found in DC conductivity: the conductivity of aligned composites was higher than the conductivity of composites with randomly distributed CNFs, especially at low filler concentration. Prasse et al. [113] also worked with a composite fabricated with epoxy resin and CNFs. CNFs showed an average diameter around 160 nm. Different filler contents were used to fabricate composites by applying a sine wave electric field of 100 V/cm at a frequency of 50 Hz during curing. The anisotropic electrical conductivities of those composites are presented in Figure 41.

The electrical properties of the composites were anisotropic. For example, for 0.5 wt.% CNFs, the network percolated only in the direction parallel to the electric field [113]. For other filler concentrations, the specific resistance (or resistivity) decreased as a function of CNF concentration, and that it was lower in the parallel direction than in the perpendicular one.

**Figure 42:**
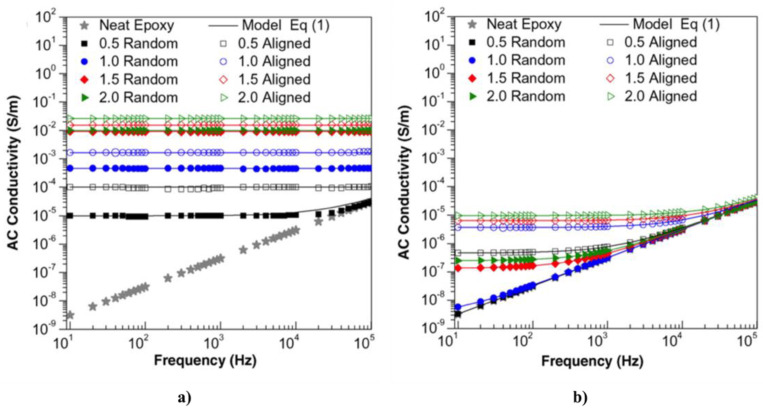
AC conductivity of epoxy nanocomposites as a function of frequency: (**a**) CNFs/epoxy nanocomposites and (**b**) GNPs/epoxy nanocomposites for various particles. Reprinted from [114], Copyright 2016, with permission from Elsevier.

Finally, Ladani et al. reported the anisotropic properties of epoxy nanocomposites reinforced by aligned nanoscale carbon [114]. They worked with two nanofillers: one-dimensional CNFs or two-dimensional GNPs. The alignment of nano-reinforcements in epoxy nanocomposites was achieved through the application of an AC before gelation and curing the epoxy resin. CNFs had a diameter of about 70–200 nm and a length of 50–200 μm, while GNPs had a thickness ranging from 1 to 20 nm and platelet diameter of ~1–50 μm. GNPs had an average thickness of ~8 nm, which equals about 18 graphene sheets based on the d-spacing of graphene, which is 0.34 nm. The epoxy nanocomposites containing 0.5, 1.0, 1.5, and 2.0 wt.% GNPs were fabricated by applying an AC of 30 V/mm at 10 kHz during the initial one-hour period of resin curing to align nanoparticles in the parallel direction to the field before resin gelation, which occurred within 1 h at 25 °C. For CNFs and GNPs, due to their form factor ratio, the longitudinal polarizability was at least an order of magnitude greater than the transverse polarizability [114]. Therefore, the interaction between the applied electric field and the induced dipole on CNFs and GNPs generates a torque, causing both nanoparticle types to rotate and align in the liquid resin. Upon gelation and subsequent curing of the resin, the aligned nanoparticles remained in place in the resulting epoxy nanocomposites. The AC conductivities for CNF/epoxy and GNP/epoxy nanocomposites are presented in Figure 42. Measurements were collected parallel to the alignment direction.

**Figure 43:**
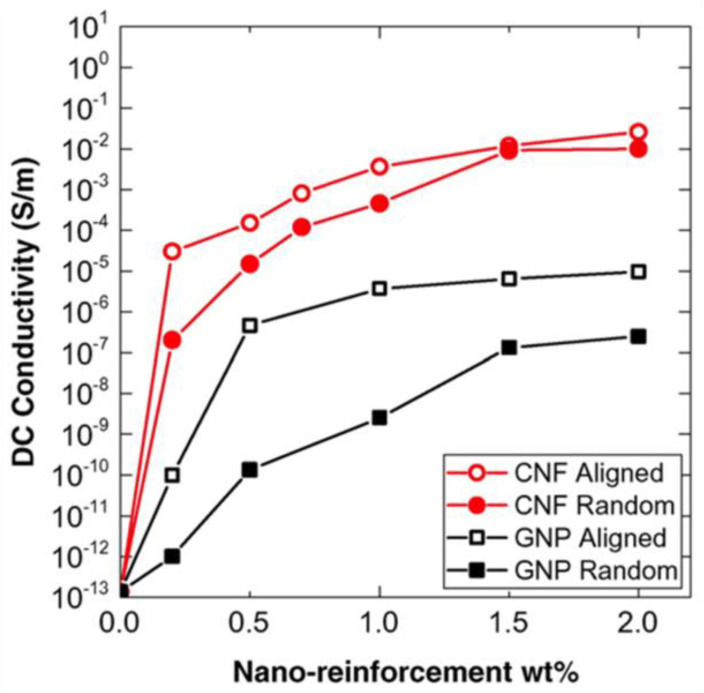
Effects of concentration and alignment of CNFs and GNPs on the DC electrical conductivities of epoxy-based nanocomposites. Reprinted from [114], Copyright 2016, with permission from Elsevier.

As discussed before, the electrical conductivities of randomly oriented and aligned nanocomposites increased with nano-reinforcement concentration, and the AC conductivity of nanocomposites with aligned nanoparticles was significantly higher than that of nanocomposites with randomly oriented nano-reinforcement [114]. The effects of concentration and alignment of CNF/epoxy and GNP/epoxy composites on DC electrical conductivities are presented in Figure 43.

Figure 43 shows that aligned composites possessed much higher electrical conductivities compared with randomly oriented composites. Additionally, the improvements in the electrical conductivities of the nanocomposites with aligned CNFs and GNPs increased by about 10 and seven orders of magnitude compared to that of the unmodified epoxy, respectively. Also, the percolation threshold of the nanocomposites containing aligned nanocarbons was about 50% lower than that of their randomly oriented counterparts. In conclusion, by applying an electric field for aligning fillers in composites, it is possible to reduce the percolation threshold, generate anisotropy, and improve conductivity.

**Figure 44:**
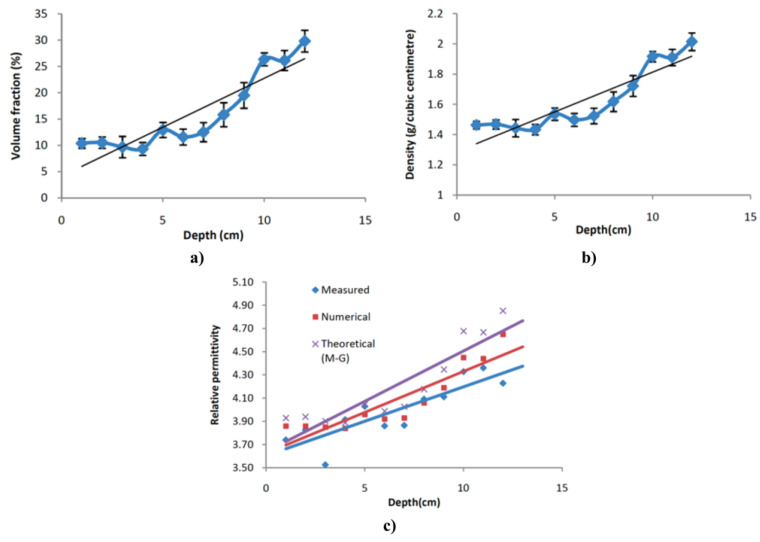
Properties vs. depth in Al_2_O_3_/epoxy composites with field-grading properties: (**a**) volume fraction, (**b**) density, and (**c**) relative permittivity. © 2021 IEEE. Reprinted, with permission, from [97].

Qasim and Gupta [97] studied the distribution of dielectric permittivity in composites for efficient electric stress control. To create an FGM, epoxy resin was used as the matrix and micro-sized alumina (Al_2_O_3_, diameter of 9 μm, density 4 g/cm^3^) was used as the filler material [97]. Gravity-assisted casting was used to obtain field-grading properties. Due to the particles denser than epoxy, they started to fall under the effect of gravity from the particle-rich upper part; however, their fall was restricted by the continuous viscosity increase of epoxy owing to curing. They reported the density, volume fraction, and permittivity of composites at different depths, as shown in Figure 44.

The volume fraction, density, and relative permittivity have a graded distribution. Then, the sedimentation technique can be used for obtaining an FGM having a linear spatial permittivity distribution [97].

**Figure 45:**
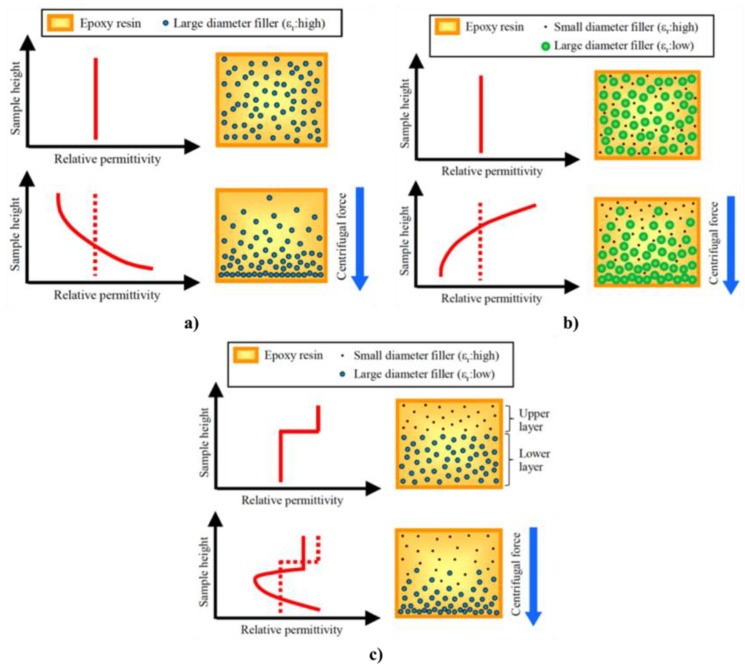
FGM Fabrication with a permittivity gradient: (**a**) GHP-FGM, (**b**) GLP-FGM, and (**c**) U-FGM. © 2021 IEEE. Reprinted, with permission, from [117].

**Figure 46:**
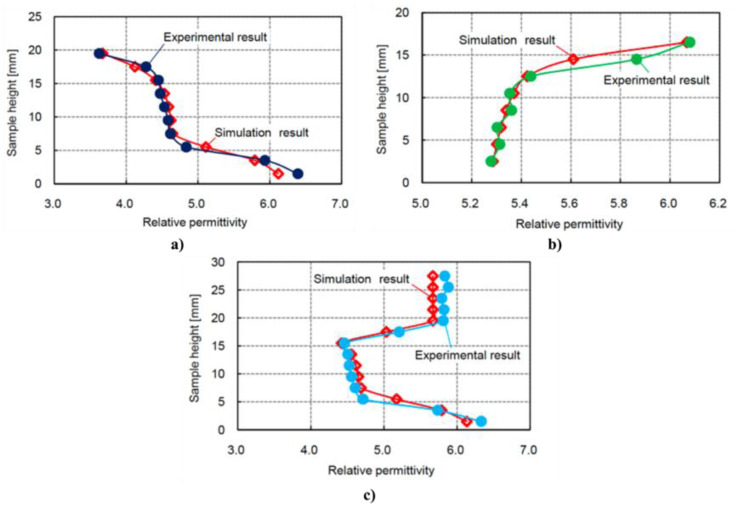
Experiments and simulation results of relative permittivity distributions in FGM: (**a**) GHP-FGM, (**b**) GLP-FGM, and (**c**) U-FGM. © 2021 IEEE. Reprinted, with permission, from [117].

Shimomura et al. [116] studied the fabrication techniques of permittivity-graded materials using particle movement simulation. The objective of their work was to develop an FGM to improve the insulation performance of solid insulators to control the electric field distribution at the solid insulator–electrode interface. For instance, in electrical systems, higher-permittivity materials around both anode and cathode electrodes relaxed the electric field at the interface. Thus, a U-shaped permittivity distribution has been developed, supported by numerical calculation, to mitigate the electric field at the electrode surface (Figure 45). The basic idea was to use fillers with different particle parameters between the upper and lower parts of the sample. Small fillers with high permittivity were filled at the upper part of the sample, and large fillers with low permittivity were filled at the lower part of the sample, as shown in Figure 45c. Under the centrifugal force, large fillers moved toward the centrifugal direction, and small fillers, less affected by the gravity, stayed at the initial location. As a result, the permittivity became low at the mid-part of the sample where the filler density reduced. Then, the permittivity increased at the lower part of the sample where the density of large fillers increased. Consequently, the U-shaped permittivity distribution was obtained using the proposed method (Figure 46c). They fabricated samples using an epoxy resin as the matrix, TiO_2_ with rutile crystal (d = 0.75 μm, ε_r_ = 114) as a small filler, and Al_2_O_3_ (d = 3.3 μm, ε_r_ = 9.3) as a large filler [116]. Kurimoto et al. [121] also investigated the feasibility of FGM fabrication with the U-shaped permittivity distribution by applying the centrifugal force.

Hayakawa et al. [117] fabricated permittivity-graded materials with the spatial dielectric permittivity distribution using centrifugal force [117]. They proposed the fabrication of an FGM with three different spatial distributions of dielectric permittivity (Figure 45): FGM with higher permittivity along the direction of the centrifugal force (GHP-FGM), FGM with lower permittivity along the direction of the centrifugal force (GLP-FGM), and FGM with U-shaped permittivity distribution (U-FGM) [117].

The experimental and simulation results for the relative permittivity of FGM composites are presented in Figure 46. Simulation results showed a good agreement with the experimental ones for the three fabrication conditions and permittivity distributions [117].

**Figure 47:**
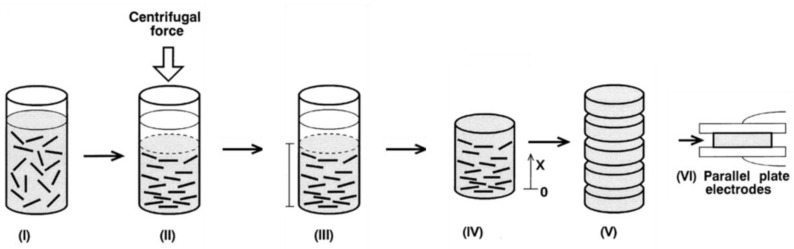
Schematic of sample preparation and measurement. (**I**) Resin and fibers before centrifugation, (**II**) direction of centrifugal force, (**III**,**VI**) sample after centrifugation, (**V**) sample cut in different specimens of 1.5 mm thickness, (**VI**) configuration for electrical characterization. Reprinted from [123], Copyright 1997, with permission from Elsevier.

The centrifugal methods were also applied to fabricate FGMs with an epoxy resin and carbon fibers [122,123,124]. For example, Funabashi [123] reported the fabrication of a composite of an epoxy resin and chopped carbon fibers (1.5 mm) coated with nickel. The process is depicted in Figure 47.

**Figure 48:**
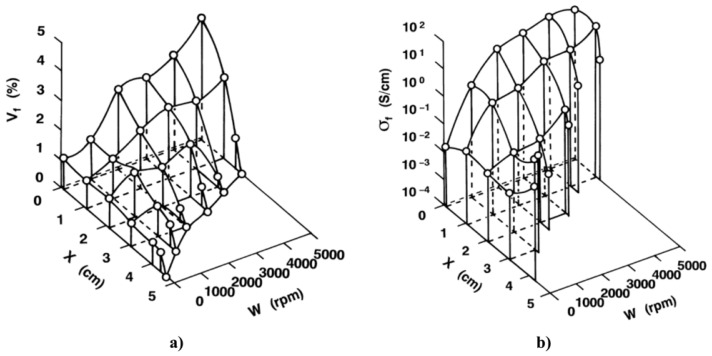
Three-dimensional relationship between (**a**) the volume fraction of carbon fibers, V_f_ (%), and (**b**) the electrical conductivity as functions of the distance X from the bottom to the center of the specimen and the maximum rotation speed, W. Reprinted from [123], Copyright 1997, with permission from Elsevier.

The gradient distribution of the volume fraction of fibers, V_f_ (%), and electrical conductivity measured perpendicularly to the centrifugal force direction (four-probe method) are presented in Figure 48. X is the distance from the bottom of the sample to the center of the specimen (Figure 47IV).

**Figure 49:**
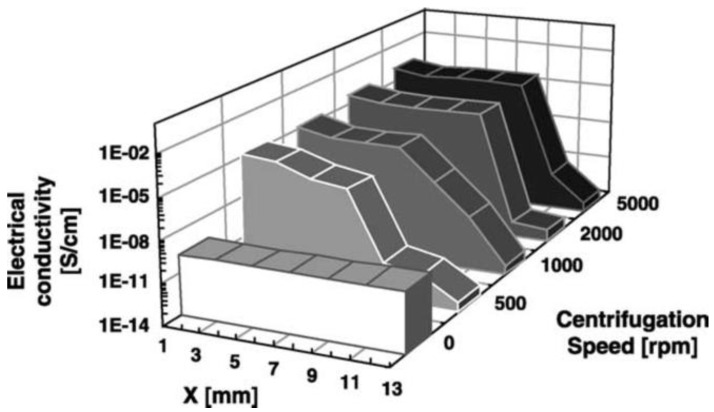
Distribution of electrical conductivity in carbon fibers/epoxy resin composites for different centrifugation speeds. Reprinted from [124], Copyright 2003, with permission from Elsevier.

As shown in Figure 48, the centrifugal force produced a gradient distribution of the carbon fiber contents in the direction of the centrifugal force. The fiber concentration gradient was affected by the magnitude of the centrifugal force and the electrical conductivity of the sample increases with increasing the rotation speed or along the distance relative to the bottom of the samples [123]. Tsotra and Friedrich [124] also fabricated an FGM using an epoxy resin and carbon fibers by centrifugation. They reported that the generated graded structure could be controlled by varying the rotation speed and the characteristics of material components, such as fiber content and aspect ratio. They used three types of pitch-based carbon fibers, an initial fiber content of 5 vol.%, and a centrifugal force under 1000 rpm. They reported a graded distribution of carbon fibers along the centrifugal force direction. The variation in the electrical conductivity of 10 vol.% medium carbon fiber/epoxy resin composite is presented in Figure 49 as a function of different rotation speeds. The bottom of the sample, where the centrifugal force was the highest, was set as point zero.

As can be observed in Figure 49, without centrifugation, the conductivity remained constant across the sample thickness. The maximum conductivity was observed at the bottom of the sample for each rotation speed. Then, the conductivity decreased rapidly, meaning that the centrifugation speed greatly influences the gradient of conductivity across the sample thickness. Lower centrifugation speeds led to a smoother decrease in electrical conductivity over distance X. At higher speeds, the samples showed a progressive conductivity decrease over a certain length before sharply returning to the insulating state of the neat matrix [124]. In conclusion, it is possible to apply the centrifugal method to generate graded filler distribution and properties in polymer composites. Final composite properties depend on process conditions and filler properties.

For solid-phase processing of FGMs, some authors have explored 3D printing. For example, Kurimoto et al. [100] fabricated a stereolithographic 3D printer with a switching function for materials between unfilled resin and alumina composite whose filler volume is 10% during the printing. Then, they fabricated a two-layered permittivity-graded material. Kurimoto [101] reported the possibility of controlling the permittivity-gradient distribution in a solid insulator via topology optimization, which is a mathematical method for optimizing the permittivity or conductivity distribution of functionally graded solid insulators in a complex insulation system. Liu et al. [102] fabricated conductivity-graded insulators using the fused-deposition-modeling 3D printing technique, which is fast and convenient, proving the potential of fabricating dielectric functionally graded materials. To investigate the feasibility of 3D printing technology in dielectric-FGM, an insulator with nonuniform conductivity was fabricated and its surface flashover characteristics were examined [104]. Compared to the uniform insulator, the surface flashover voltage of the nonuniform insulators was improved by 23% in SF_6_ and 20% in vacuum [104]. Thus, they concluded that 3D printing of FGM has potential applications in insulation designs [104].

## 7. Current Applications of Nonlinear Polymer Composites in HV Engineering

**Figure 50:**
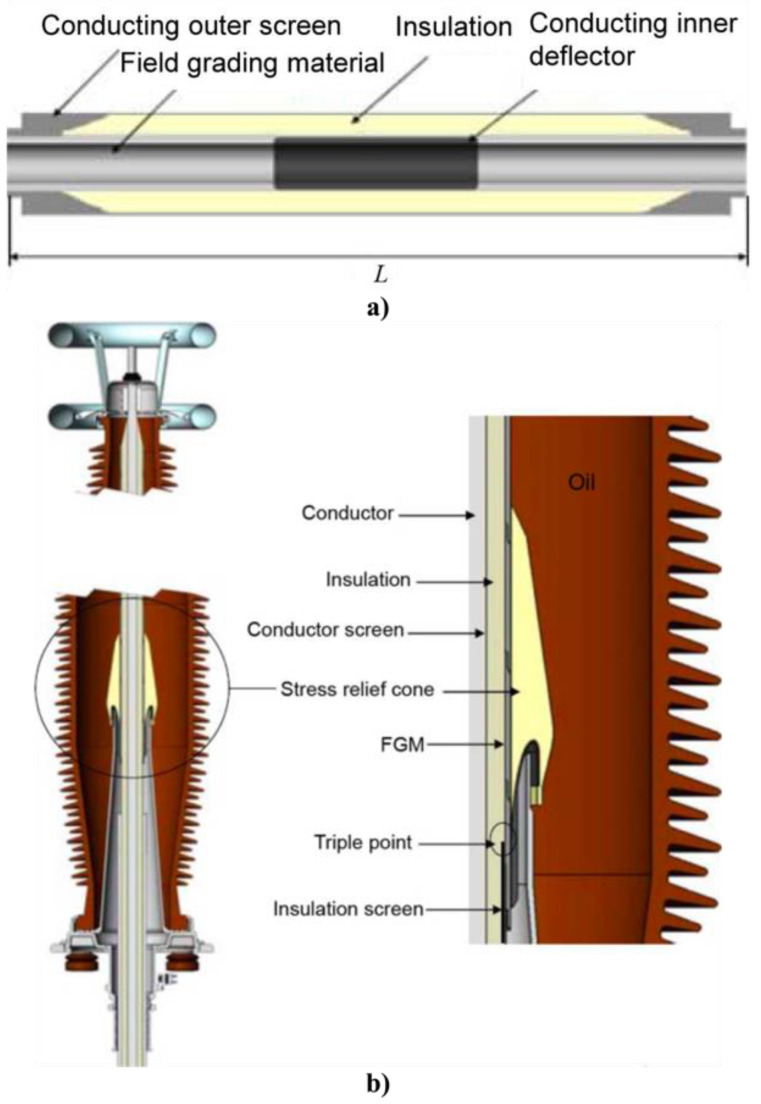
Cable accessories with field-grading materials: (**a**) typical prefabricated joint design and (**b**) HVDC cable termination. © 2021 IEEE. Reprinted, with permission, from [37].

The composite materials with nonlinear properties have an important role in HV systems as they can reduce the electric field stress, which is the origin of electrical aging and insulation failure. For example, in HV motor windings, it is possible to find nonlinear resistance tapes as stress grading materials [21]. Materials with nonlinear dielectric behaviors are applied to rotating electrical machines for the control of the electrical field strength in the insulation system of stator end windings [28]. Nonlinear conductive materials enable the grading of electric-field distributions in polymeric outdoor insulators, particularly near the high-voltage and ground terminals [125]. It is also possible to find nonlinear materials in cable accessories [22,36,61], such as cable joints and HVDC cable terminations (Figure 50) [37,126].

**Figure 51:**
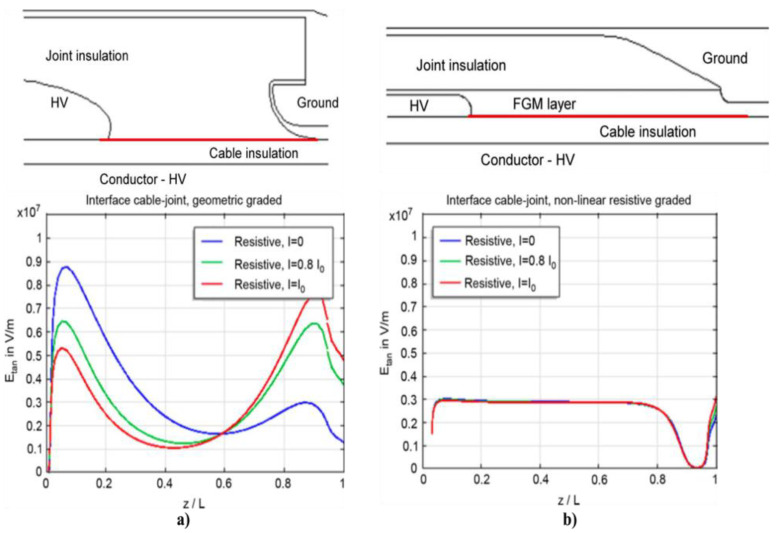
Tangential electric field along the cable–joint interface under DC applied voltage: (**a**) geometrically graded joint (**top**) and (**b**) nonlinear resistive graded joint (**top**). © 2021 IEEE. Reprinted, with permission, from [37].

Ghorbani et al. reported the difference between the tangential electric field along the cable–joint interface for a joint with geometric and nonlinear resistive grading (see Figure 51) [37].

For the geometrically graded system (Figure 51a), a major part of the voltage drop between the HV and ground side is typically confined to a small interface, and the electric field maxima close to the electrodes depend on the current load. In FGMs (Figure 51b), one can observe a flattened resistive tangential field distribution along the cable–joint interface. Moreover, this distribution is robust against variations in the current load [37].

**Figure 52:**
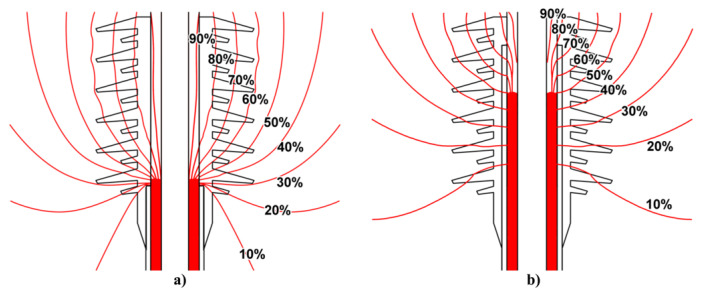
Equipotential line distribution under applied voltage (100 kV, AC): (**a**) EPDM-graded cable tube ($\varepsilon_{r}$ = constant) and (**b**) nonlinear composite-graded cable tube. © 2021 IEEE. Reprinted, with permission, from [23].

Another example is the control of the field distribution at a cable end by placing a ZnO stress control tube over the screen cut area. Thus, the electrical stress is limited to a certain level (Figure 4b, curve b). This level does not change even at higher voltages (Figure 4b, curve c). Boucher et al. [23] studied by finite element simulation a shielded cable termination. The nonlinear material tube was applied at the end of the shield as an extension and on the insulation as a sleeve. The equipotential lines of two different cable terminations are presented in Figure 52 [23].

The concentration of equipotential lines remains important, especially near the end of the shield in the case of the standard (EPDM) cable termination. For nonlinear coating, the lines are spread throughout the layer, reducing the electric field peak [23].

**Figure 53:**
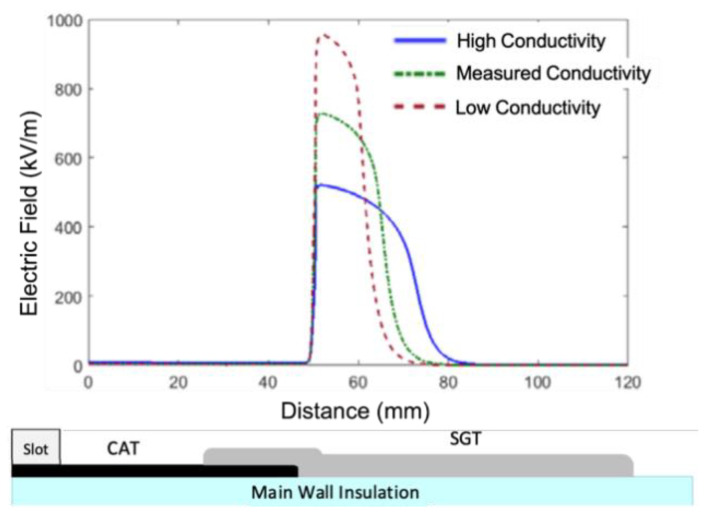
Electric field distribution along the stress-grading system of an inverter-fed rotating machine. © 2021 IEEE. Reprinted, with permission, from [40].

Naeini et al. [40] simulated the effect of tape conductivity on the electric field distribution in a stress-grading system of an inverter-fed rotating machine. The main parts of this system consisted of a semiconductive armor tape (CAT) and a stress-grading tape (SGT). CAT, commonly made of CB embedded in a fiberglass tape, is used in the corona suppression of form-wound coils, and SGT is used to prevent PD at the end of the CAT in the overhang region of a rotating machine. The geometry of the stress-grading system used in the FEM is presented in Figure 53. The results of the simulation with three different levels of SGT conductivities are also presented [40].

The increment of the SGT conductivity decreased the electric field. They reported that the conductivity of SGT depended on the electric field and was high at the end of the CAT, where the electric field was very high, and gradually decreased along the SGT. Therefore, it makes the electric field at the end of the CAT more uniform. Umemoto et al. [41] also reported the use of a nonlinear grading tape to control the field in a stator bar from large rotating machines.

**Figure 54:**
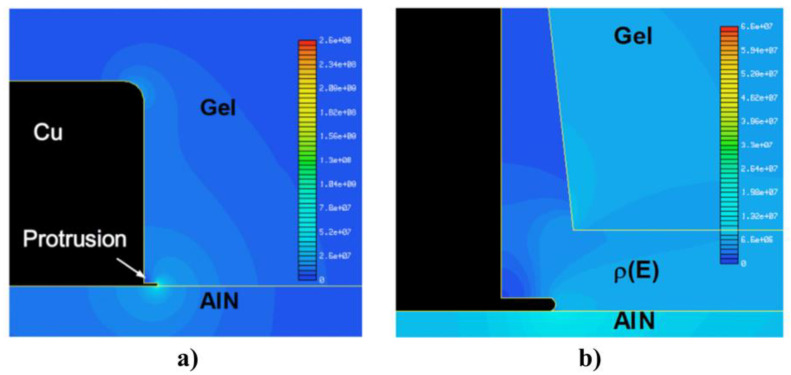
Electrical field calculated by finite element simulation. Material (**a**) without and (**b**) with a nonlinear field-grading layer. © 2021 IEEE. Reprinted, with permission, from [33].

Donzel and Schuderer [33] reported the application of nonlinear resistive electric field control for high-power modules with insulated-gate bipolar transistors. As discussed before, the electric field at the edges of the substrate metallization increases accordingly and can exceed the dielectric strength of the gel, commonly used for encapsulation, leading to PDs or even insulation breakdown. They studied how the field concentration could be significantly reduced by applying a functional coating with suitable nonlinear resistive characteristics by finite element simulations. Their results are presented in Figure 54 [33].

In Figure 54a, the maximum electric field inside the gel (without the nonlinear resistive layer) was found at the protrusion tip (2.6 × 10^8^ V/m). Figure 54b shows that the electric field at the protrusion reduced to 7 × 10^6^ V/m because of the equipotential line spreading that reduced the field nonuniformity. Then, with a nonlinear resistive coating at the metallization edges, field peaks due to protrusions or other defects were efficiently mitigated [33].

**Figure 55:**
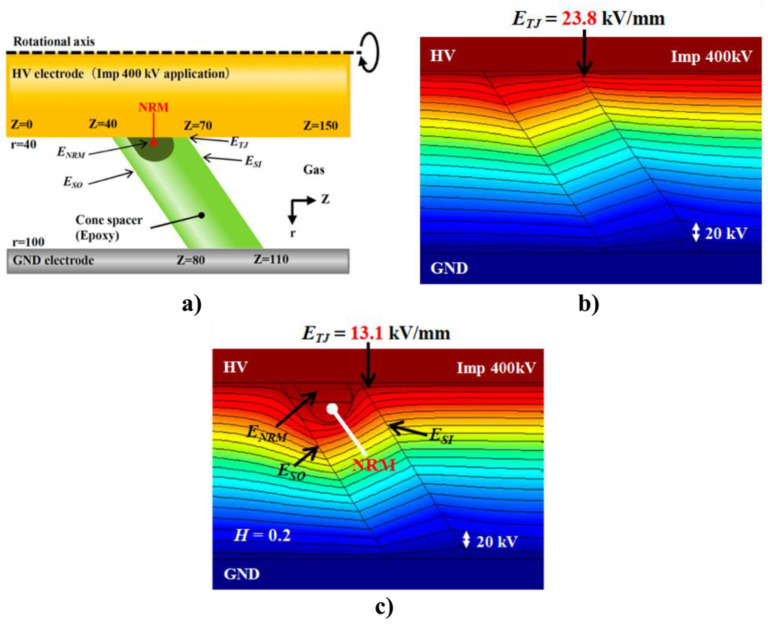
Gas-insulated switchgears using nonlinear resistive materials: (**a**) model, (**b**) distribution of electric field without and (**c**) with nonlinear resistive material (*E* = 1000 V/mm). © 2021 IEEE. Reprinted, with permission, from [47].

Other applications of electric field-grading materials have been reported for gas-insulated switchgears. For example, Matsuoka et al. [47] worked with the model shown in Figure 55a, where a material with nonlinear resistive material was applied to a cone spacer for gas-insulated switchgears.

**Figure 56:**
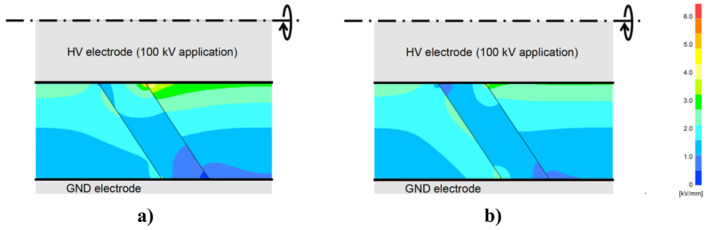
Electric field distribution for a cone-type spacer. (**a**) Uniform spacer, (**b**) FGM spacer. © 2021 IEEE. Reprinted, with permission, from [117].

The comparison of the electric field at the triple junction (*E_TJ_*) between materials with and without the nonlinear resistive material is shown in Figure 55. Thus, the maximum electric field was reduced by 45% using nonlinear resistive materials. Hayakawar et al. [117] reported the simulation of permittivity-graded materials for electric field grading of gas-insulated power apparatus. They worked with the simulation of a cone-type spacer arranged in a coaxial configuration between HV (100 kV) and ground (GND) electrodes. The comparison of a uniform spacer with uniform permittivity ($\varepsilon_{r}$ = 6.0) and an FGM spacer with a relative permittivity proportional to the inverse of the radius (1/r) from $\varepsilon_{r}$ = 9.0 to $\varepsilon_{r}$ = 3.0 is presented in Figure 56 [117].

**Figure 57:**
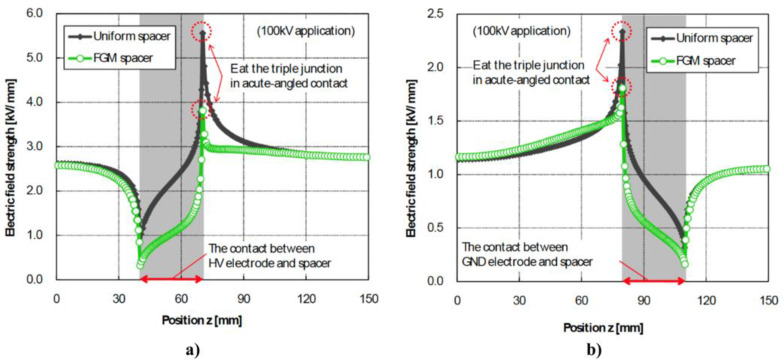
Electric field distribution on HV and GND electrode surfaces. Electric field strength on (**a**) HV electrode surface and (**b**) GND electrode surface. © 2021 IEEE. Reprinted, with permission, from [121].

Figura 56 shows the electric field distribution around the uniform and FGM spacers. Due to the application of an FGM, the electric field distribution around the spacer is more uniform, and the electric field at the triple junction is relaxed [117,121,127,128]. Kurimoto et al. [121] reported that by the introduction of the FGM spacer, the electric field distribution in the spacer was improved. They reported the electric field distribution along the surface of the HV and GND electrodes (Figure 57). The electric field strengths on both electrode surfaces in contact with solid insulators were reduced by the introduction of FGM spacers, and the FGM spacer reduced the intensified field strength at triple junctions at z = 70 mm in Figure 57a and z = 80 mm in Figure 57b [121].

We conclude that FGMs with nonlinear conductivity or permittivity can play an important role in HV applications by controlling the electric-field nonuniformity and reducing electrical stress in insulating materials [129,130,131]. By reducing stress concentration in electrical systems, overall insulation degradation can be largely delayed.

## 8. Concluding Remarks

In high-voltage applications, electric stress is one of the factors that influences the rate of aging of systems. When high electric stress is applied to most polymers, charges are induced and accumulated within the polymers and the insulation degrades because of space charge accumulation, partial discharge, and electrical treeing across the entire material, which may cause the material to break down. Therefore, it is necessary to control the strength of the electric field. The aim is to achieve the weakest possible electric field at a fixed system voltage. 

There are two strategies for controlling electrical stress: capacitive control and resistive stress control. In resistive stress control, the application of nonlinear stress control is a good alternative. Nonlinear stress control employs materials whose resistivity varies with the applied voltage. These materials are obtained from a base material, which is usually an insulating polymer, with an additive that provides nonlinear functionalities. Nonlinearity in composites originates from two sources: particle–particle contacts and intrinsic properties of filler particles. The conductivity of the final composite depends on the filler type, its physicochemical characteristics, size and shape, and interaction with the matrix. 

ZnO and SiC are some of the most commonly used filler materials for nonlinear conduction. The nonlinear conductivity of composites strongly depends on the filler concentration. The switching of field decreases as the filler concentration increases because the nonlinear conductivity of composites containing fillers is highly determined by the conduction paths formed by the fillers. In addition, composites with a higher amount of filler present sooner nonlinear electrical properties. Nonlinear materials can also be obtained using graphene oxide as a filler, and the nonlinearity of the composite can be tuned by modulating the reduction degree of graphene oxide. Another alternative method is using composites with multiple particle types, different sizes, morphology, or nature. Results show that combining different fillers helps to tune the final electrical properties of composites and results in different nonlinear conduction properties depending on the application specifications. 

On the contrary, it is possible to control the distribution of the composite properties by controlling the filler distribution. The centrifugal method, electrical deposition, and 3D printing are suitable techniques for controlling the final properties of composite materials. For example, when an electric field is applied during the material fabrication, it is possible to induce the alignment and orientation of particles, including CNTs, carbon fibers, and graphene nanoplatelets. The electrical properties of the obtained composites are anisotropic and depend on the electric field characteristics. The centrifugal method can be used to fabricate functionally graded materials because the centrifugal force causes the gradient distribution of the fillers in its direction. The filer concentration gradient is affected by the magnitude of the centrifugal force and the size and density of the filler. The properties of the composite depend on this distribution. 

Finally, we present the current applications of nonlinear polymer composites in high-voltage engineering herein. Composite materials with nonlinear properties play an important role in high-voltage systems as they can control the nonuniformity of the electric field and reduce electrical stress in insulating materials. By reducing the stress concentration in electrical systems, space charge accumulation, PD, and electrical treeing can be efficiently reduced and insulation degradation can be achieved.

From this perspective, the strategies for electrical-stress control depend on the applications and some features. Fabricating composite materials using nonlinear fillers remains an interesting alternative that can be complemented with one of the three great fabrication alternative methods: centrifugation, orientation by an electric field, and 3D printing. Combining these two resources opens up the possibility of enhancing the reinforcement of materials in the weakest areas, thereby extending their lifespan.

## Figures and Tables

**Table 1:** Characteristics of ZnO fillers used in nonlinear composites.

Matrix	Filler	Characteristic	Size	Ref.
Polyester	ZnO	-	~6 μm (granule size)	[43,44]
Epoxy resin	ZnO	Undoped, 99.9% purity	1 μm	[45]
	ZnO	99 mol % ZnO + 1 mol % K_2_CO_3_	50–300 μm	[46]
	Microvaristor	-	21.3–38.2 μm	[47]
Low-density polyethylene	ZnO	-	1 μm (diameter)	[48]
	ZnO	Nanoparticles	~49 nm	[49]
	ZnO	Boundary formers (Bi_2_O_3_ + Sb_2_O_3_). Conductivity enhancers (CoO + MnO). Shape (almost spherical)	0.1–1 μm (diameter)	[50]
Polyethylene	ZnO	Varistor powder, polycrystalline filler, spherical shape	60–160 μm	[51]
Silicone rubber	ZnO	Microvaristor, spherical, and irregular particles	50–150 μm for spherical (diameter). 20–125 μm for irregular.	[52]
	ZnO	Microvaristor + other particles	60.5 μm (Average diameter)	[53]
	ZnO	Varistor, 95 mol % ZnO + 1.0 mol % Bi_2_O_3_ + 0.5 mol % MnO_2_ + 1.0 mol % Co_2_O_3_ + 0.4 mol % Cr_2_O_3_ + 1 mol % Sb_2_O_3_ + 1.0 mol % SiO_2_ + 0.1 mol % Al_2_O_3_	50–150 μm (microspheres)	[54]
	ZnO		120 μm (microspheres) 9.5 μm (grains)	[55]
	ZnO		Spherical, grain size in the range of 10–15, 5–8, and 1–4 μm.	[56]
	ZnO	Microvaristor (spherical)	15–100 μm	[57]
Ethylene propylene diene monomer	ZnO	Nanoparticles modified on SnF_2_ or SnCl_2_	63 nm	[58]

**Table 2:** Characteristics of SiC fillers used in nonlinear composites.

Matrix	Filler	Characteristic	Size	Ref.
Silicone rubber	SiC	Spherical conformed by α-SiC hexagonal crystal	Diameter of 0.45–0.50 μm	[59,60]
EPDM	SiC	β-crystal	Diameter of 0.50 μm	[61]
	SiC	Green, n-type, with N	22.8, 9.3, 3.0, and 0.7 μm	[62]
x	SiC	Black, purity of 99.9%	From 2.06 to 50.6 µm	[63]
x	SiC	Green, n-type, with 5 × 10^24^/m^3^ of N and 4–6 × 10^24^/m^3^ of Al	Grain diameters of 22.8, 9.3, and 3.0 µm	[64]
		Black, p-type, with ≈10^26^/m^3^ of Al	Grain diameters of 9.3 µm	
x	SiC	Undoping, gray	125, 63, 36.5, 6.5 μm	[65]
		Doping with Al, p-type, black		
		Doping with N, n-type, green		

**Table 3:** Characteristics of carbon allotrope fillers used in nonlinear composites.

Matrix	Filler	Characteristic	Size	Ref.
Polyethylene	Carbon black (CB)	Surface area of 19 m^2^/g	90 nm	[66]
High-density polyethylene	CB	Surface area of 230 m^2^/g	50 nm (average)	[67]
	Carbon nanofiber	-	Diameter of 100–200 nm	[68]
-	Single wall carbon nanotubes (SWCNT)	-	Diameter of 2 nm Length 300–1100 nm	[69]
-	SWCNT	Produced by electric-arc-discharge	Diameter of 1.2–1.5 nm	[70]
Polystyrene	SWCNT	70% pure with some Ni and Yt catalysts residue	Diameter of 1.3 nm	[71]
Polyurethane	SWCNT			[71]
Polydimethylsiloxane rubber	multi-walled carbon nanotubes (MWCNT)	Grown by chemical vapor deposition (CVD)	Diameters around 10 nm Lengths around 10 μm	[72]
Epoxy resin	Graphite nanosheet	The number of sheets in the platelets is 150	Thickness of 50 nm Diameter of 12 μm	[73]
LDPE	Graphene nanoplatelets	Surface area of 120–160 m^2^/g Density of 2.2 g/cm^3^	Diameter of 25 μm, Thickness of 6–8 nm	[74]
Poly(dimethyl siloxane)	Graphene oxide (GO)	Mostly monolayer GO Study of the effect of thermal reduction	Lateral dimension of 500 nm, thickness of 1.1 nm	[75]
Silicone rubber	GO	Study of the effect of thermal reduction	-	[76]

**Table 4:** Nonlinear conduction properties of composites involving ZnO as a filler.

Matrix	Filler (vol.%)	*E_b_* (V/mm)	α	Ref.
PE	20	600–800	9	[51]
	35	200	9	
	45	180	9	
	50	190	9	
LLDPE	14.10	X	X	[50]
	24.71	924	32	
	32.99	589	31	
	39.63	427	22	
	45.01	263	18	

**Table 5:** Nonlinear properties of composites involving ZnO as a filler: effect of filler size.

Matrix	Filler	Content (vol.%)	Size (μm)	*E_b_* (V/mm)	α	Ref.
Epoxy resin	ZnO	20	50–100	500	14.98	[46]
			100–150	120	15.56	
			150–200	70	15.82	
			200–300	50	16.08	
Silicone rubber	ZnO microspherical	39	50–75	826.5	10.2	[54]
			75–100	780.8	10.3	
			100–125	575.1	10.0	
			125–150	506.7	10.8	
		46.5	50–75	522.8	10.2	
			75–100	419.2	12.6	
			100–125	408.0	12.7	
			125–150	329.4	17.5	
	ZnO spherical	46.5	50 to 75	600	11	[52]
			75 to 100	541	11	
			100 to 125	458	12	
			125 to 150	325	13	
	ZnO irregular	46.5	20 to 35	1731	15	
			35 to 50	1538	15	
			50 to 75	1288	14	
			75 to 125	1331	15	
	ZnO spherical (75–125 μm)	39	Grain size 10–15 μm, (Z1)	360	12.3	[56]
		46		321	15.6	
		100 (filler)		250	21.4	
		39	Grain size 5–8 μm, (Z2)	523	14.9	
		46		471	15.6	
		100 (filler)		463	22.6	
		39	Grain size 1–4 μm, (Z3)	1563	14.5	
		46		1462	16.2	
		100 (filler)		1440	22.5	

**Table 6:** Nonlinear parameters of ZnO varistors modified with Y_2_O_3_ [79].

Sample	Y_2_O_3_ (mol %)	Grain size (μm)	*E_b_* (V/mm)	α
ZY000	0	10.13	315.6	35.1
ZY050	0.50	7.22	402.2	29.8
ZY075	0.75	6.85	505.5	21.6
ZY100	1.00	6.03	737.4	15.4

**Table 7:** Nonlinear properties of composites involving SiC as a filler: effect of filler concentration [59,60].

Matrix	Filler	Size (μm)	Content (wt.%)	*E_b_* (kV/mm)	α	Ref.
Silicone rubber	SiC	0.45	0	20	x	[59,60]
			10	17	x	
			30	3.0–3.8	0.97	
			50	1.4	1.04	
			100	0.9	1.07	
EPDM	SiC	0.5	10	12	x	[61]
			30	9	x	
			50	7	x	

**Table 8:** Switching field for GNs/epoxy composites [73].

GN (vol.%)	*E_b_* (V/cm)
1.800	6.65
2.055	7.95
2.356	8.69
2.555	10.30
2.704	10.54
3.051	12.10

**Table 9:** Material compositions and switching field for multifunctional silicon rubber composites [53].

Material (vol.%)	SiR P401/20 (vol.%)	ZnO (vol.%)	Al(OH)_3_ (vol.%)	SiR R570/50 (vol.%)	Oil (vol.%)	Cross-Linking Agent (vol.%)	*E_b_* (V/m)
FGM01	62	38	0	0	0	0	3 × 10^4^
FGM02	52	20	25	0	1	2	1 × 10^6^
FGM03	75	20	0	5	0	0	1 × 10^6^
FGM04	47	20	25	5	1	2	1 × 10^6^
FGM05	77	20 (Ag)	0	0	1	2	1 × 10^5^
FGM06	49	30	17	0	1	3	1 × 10^4^
SiR	100	0	0	0	0	0	N/A

**Table 10:** Composition, nomenclature, and nonlinear coefficient of epoxy-based composites with different fillers [95].

Sequence Number	Sample	Nano-ZnOwt.%	Micro-ZnOwt.%	Nano-SiCwt.%	*E_b_* (kV/mm)	α	Sample Category
1	EP	-	-	-	5.27	0.12	EP
2	ZnO#1Q	1	-	-	6.40	0.52	ZnO/EP nanocomposites
3	ZnO#3Q	3	-	-	5.84	0.65	
4	ZnO#5Q	5	-	-	2.32	0.64	
5	ZnO#1Z	-	1	-	3.15	0.46	ZnO/EP microcomposites
6	ZnO#3Z	-	3	-	2.90	0.78	
7	ZnO#5Z	-	5	-	2.16	0.98	
8	SiC#1	-	-	1	5.39	0.58	SiC/EP nanocomposites
9	SiC#3	-	-	3	2.23	1.70	
10	SiC#5	-	-	5	1.14	1.69	
11	ZnO#1Q&SiC#4	1	-	4	2.45	2.32	SiC/ZnO/EP nanocomposites
12	ZnO#2Q&SiC#3	2	-	3	4.69	2.96	
13	ZnO#3Q&SiC#2	3	-	2	6.28	2.05	
14	ZnO#4Q&SiC#1	4	-	1	6.26	1.44	
15	ZnO#1Z&SiC#4	-	1	4	X	2.57	SiC/ZnO/EP micro/nanocomposites
16	ZnO#2Z&SiC#3	-	2	3	1.51	3.50	
17	ZnO#3Z&SiC#2	-	3	2	1.52	2.31	
18	ZnO#4Z&SiC#1	-	4	1	1.56	NA	
